# Recent advances in GelMA hydrogel transplantation for musculoskeletal disorders and related disease treatment

**DOI:** 10.7150/thno.80615

**Published:** 2023-03-27

**Authors:** Bin Lv, Li Lu, Liangcong Hu, Peng Cheng, Yiqiang Hu, Xudong Xie, Guandong Dai, Bobin Mi, Xin Liu, Guohui Liu

**Affiliations:** 1 Department of Orthopedics, Union Hospital, Tongji Medical College, Huazhong University of Science and Technology, Wuhan, 430030 P.R. China.; 2 Pingshan District People's Hospital of Shenzhen, Pingshan General Hospital of Southern Medical University, Shenzhen, 518118 P.R. China.; 3 Third School of Clinical Medicine, Affiliated Hospital of Integrated Traditional Chinese and Western Medicine, Nanjing University of Chinese Medicine, Nanjing, 210028 P.R. China.

**Keywords:** tissue engineering, hydrogel, nanomaterials, musculoskeletal disorders, bone

## Abstract

Increasing data reveals that gelatin that has been methacrylated is involved in a variety of physiologic processes that are important for therapeutic interventions. Gelatin methacryloyl (GelMA) hydrogel is a highly attractive hydrogels-based bioink because of its good biocompatibility, low cost, and photo-cross-linking structure that is useful for cell survivability and cell monitoring. Methacrylated gelatin (GelMA) has established itself as a typical hydrogel composition with extensive biomedical applications. Recent advances in GelMA have focused on integrating them with bioactive and functional nanomaterials, with the goal of improving GelMA's physical, chemical, and biological properties. GelMA's ability to modify characteristics due to the synthesis technique also makes it a good choice for soft and hard tissues. GelMA has been established to become an independent or supplementary technology for musculoskeletal problems. Here, we systematically review mechanism-of-action, therapeutic uses, and challenges and future direction of GelMA in musculoskeletal disorders. We give an overview of GelMA nanocomposite for different applications in musculoskeletal disorders, such as osteoarthritis, intervertebral disc degeneration, bone regeneration, tendon disorders and so on.

## Introduction

Musculoskeletal tissue comprises muscles, bones, cartilage, tendons, and ligaments representing more than 40% of body mass and providing shape and structural support for the body. In recent years, there has been a marked rise in debilitating diseases related to musculoskeletal tissue due to increased life expectancy 1. Indeed, one-third of the population suffer from chronic pain associated with musculoskeletal disease, such as fractures, osteoarthritis, tendonitis, and rheumatoid arthritis, which severely affects the patients' physical and mental health, leading to reduced work efficiency and segregation of social activities 2. Tissue engineering is increasingly implemented to solve the critical difficulties of musculoskeletal diseases, including bone, cartilage, tendon/ligament, and skeletal muscle. At present, autografts, allografts, and xenografts related to tissue engineering have been used for musculoskeletal disorder treatment, but they have associated complication risks, including immune rejection, disease transmission, and infection. Currently there are many synthetic materials used for bone scaffolds, mainly inorganic materials such as bioceramics, calcium carbonate, bioactive glass and titanium alloys, which, although they have good biocompatibility and osteoconductivity, are fragile and have poor flexibility 3. For the more easily synthesised organic materials such as polylactic acid and polyglycolic acid, they have better mechanical properties and biocompatibility but lack the biological activity to induce good regeneration of osteochondral bone 4. Therefore, it is vital to develop alternative scaffolds that can effectively modulate the multiple cellular processes including cell proliferation, differentiation, migration, homing, etc., and can also load target cytokines to improve targeting ability towards specific tissues 5.

Among the various developed bioscaffolds, hydrogels have received considerable attention due to their good hydrophilicity, tunable physicochemical properties, and extracellular matrix-mimicking properties 6. Currently, hydrogels based on both naturally-derived and synthetic polymers have been widely used in bioscaffolds through physical and chemical modification to improve cell behavior and activate molecular signaling. Notably, compared with synthetic polymers, natural polymer-based hydrogels exhibit superior biocompatibility, controllable biodegradability, superior bioactivity, and low immunogenicity 7. However, its poor mechanical properties and mismatched degradation rates pose challenges for clinical translation. Gelatin, unlike PEG, is a natural polymer hydrolyzed from collagen. It is a common structural protein component in the extracellular matrix (ECM) of cartilage, bones, tendons, and ligaments. However, gelatin's lack of stable triple helical structure leads to its poor mechanical properties and uncontrollable degradation. Therefore, it has become a trend to improve gelatin's properties by chemical cross-linking and imparting certain functionality. Gelatin modified with methacryloyl (MA) pendant groups (GelMA) has been increasingly developed due to its tunable biological functionality and enhanced mechanical properties to a reasonable extent 8. More importantly, the gelatin modified with methacrylic anhydride (MA) involves amino acid residues with a molar ratio of less than 5%, so functional amino acid sequences, such as RGD sequences, are not significantly affected 9. In addition to interactions with cells, GelMA plays a key role in tissue morphogenesis in vitro and* in vivo*. In situ injection of formable biodegradable hydrogels, on the other hand, has been shown to be a minimally invasive method of treating musculoskeletal problems, especially the hydrogels exhibit flexibility, porosity, and mimic the natural extracellular matrix (ECM) capacity of the microenvironment. GelMA endows scaffolds with the required structural and biological properties for drug delivery, tissue regeneration, and cell culture, based on its crosslinking to form hydrogels with biocompatibility, tenability, and biomimicry. These factors make them suitable for optimal drug delivery, tissue integration, and regeneration, especially for treatment of musculoskeletal disorders. This review was undertaken to summarize the current application of GelMA as a material and drug or gene delivery carrier in the field of musculoskeletal disorder. Finally, we discuss the challenges and future prospects of GelMA, hoping to facilitate the advances of GelMA in the biomedical areas.

## An overview of GelMA

GelMA is a gelatin derivative whose precursor natural material, gelatin, is mainly a denatured collagen product 10,11. Gelatin exhibits better solubility and lower antigenicity 12. The presence of Arg-Gly-Asp (RGD) sequences promotes the biological interaction between cells and scaffolds, and thus an attractive biomaterial. In addition, presence of matrix metalloproteinase (MMP)-sensitive targeting sequences makes cell remodeling easier implementable, and thus an attractive biomaterial 13,14. The hydrolysis process destabilizes the tertiary structure of collagen, minimizing structural variation due to different sources.

Gelatin solutions also have unique low-temperature gelation properties, which can form physically cross-linked hydrogels 15. However, uncontrollable degradation rate and poor mechanical properties restrict its application range, especially in treating weight-bearing skeletal diseases 16,17. Therefore, it becomes necessary to apply chemical cross-linking reactions to prepare appropriate hydrogel materials 13. In comparison to other known hydrogel-forming biomaterials, gelatin modified by methacryloyl (methacrylamide and methacrylate) side groups (GelMA) has met the requirements of mechanical tenability and biofunctionality 18. GelMA is synthesized by the direct reaction of gelatin with methacrylic anhydride (MA) in phosphate buffer at 50 °C. This modification modify gelatin by substituting methacryloyl substitution groups and hydroxyl groups of the amino acid residues onto the reactive amine 19. Gelatin is rapidly polymerized in the presence of UV light and a photocuring agent due to the introduction of MA 20.

Methacrylic anhydride (Ma) has been used to modify gelatin by substituting methacryloyl substitution groups onto the reactive amine so as to obtain photoreactive gelatin methacrylate (GelMa), which can be crosslinked when exposed to UV light and a photocuring agent.

Compared to other methods, photo polymerization displays numerous benefits, such as injectability, rapid gelation, improved mechanical properties, suitability for customized bioprinting along with easy incorporation with various cell types. However, the free radicals generated during crosslinking can attack cell membranes and result in cell death 21. This effect depends on the source of radiation and intensity of UV light. Studies have also showed that photo crosslinking at milder conditions is biocompatible, which can be easily tuned by reducing the intensity from UV source and amount of photoinitiator used. It has also been reported that a high density of methacryloyl groups can have protective effects for incorporated cells.

The mechanical properties of hydrogels can modulate the biological response of cells. Studies have found that the viscoelasticity of hydrogels promotes the proliferation and diffusion of mesenchymal stem cells (MSCs) 22. Moreover, it also enhances the mechanotransduction of cells and the osteogenic differentiation of stem cells 22,23. Due to its excellent mechanical properties and biological activity, GelMA can produce significant benign induction of osteoblasts, chondrocytes and stem cells. It provides a good environment for cell adhesion, proliferation and differentiation, and modification of the mechanical properties of GelMA by light, amongst others, can stimulate cellular responses for therapeutic needs in different diseases. Mechanical stimulation is an effective means of promoting cartilage matrix secretion, which can affect the chondrocyte phenotype and alter the expression of extracellular matrix components, thereby enhancing the mechanical properties of the tissue building cartilage 24. Zeng et al. 25 developed GelMA hydrogels reinforced with bacterial nanocellulose (BNC), which significantly improved the mechanical properties of the hydrogels, enhanced cell migration and provided a mechanical environment for cartilage formation. Li et al. 26 found that GelMA hydrogels with high stiffness (29.9 kPa) could better maintain cartilage development by preparing hydrogels with different stiffness. In addition, GelMA-based scaffolds constructed by 3D printing increased the viability of human bone marrow mesenchymal stem cells (hBMMSCs) and enhanced the expression of Runx2 27. It can be seen that the regeneration of tissues such as bone and cartilage can be effectively achieved by constructing GelMA hydrogels with appropriate mechanical properties.

The implantation of biomaterials into the body induces an immune response in the host, which if caused by a severe or sustained inflammatory response can lead to erosion of the extracellular matrix, the death of key cells and ultimately the failure of tissue repair. Amongst all immune cells, macrophages are one of the most important cells in the process of inducing an immune response to biomaterials. Excellent biomaterials based on GelMA hydrogels, when injected into the body, should have good immunomodulatory properties, including promoting the M2 polarisation of macrophages, inhibiting M1 polarisation, regulating the production of T cells and reducing the inflammatory response, thereby promoting the regeneration of blood vessels, bone, cartilage, nerves, skin and other tissues 28-32. For instance, Li et al. 33 constructed an injectable photocrosslinkable porous GelMA/serine protein glycidyl methacrylate (SilMA) hydrogel encapsulating gingival tissue-derived MSCs (GMSCs) that promoted M2 polarization while inhibiting M1 polarization *in vitro*. To enhance the anti-inflammatory properties, Li et al. 34 blended a potent anti-inflammatory agent, curcumin (Cur), into the GelMA hydrogel. This anti-inflammatory platform not only exerts excellent anti-inflammatory effects, but is also biocompatible and shows good ability to promote cartilage regeneration *in vitro*. In order to modulate the immunological properties of macrophages, Wen et al. 30 combined a nanoporous poly (propylene-co-ethylene-glycolide) (PLGA) microsphere delivery system with GelMA hydrogels to continuously release soy lecithin (SL) and IL-4 complexes (SL/IL-4), an approach that inhibits excessive local inflammatory counter-reactions while promoting vascular maturation and ultimately rapid tissue repair. Optimising the immunomodulatory capacity of GelMA in vivo is an important component of regenerative medicine and could provide new ideas for future clinical practice.

It is worth noting that the chemical modification of gelatin by methacrylic anhydride (MA) only involves amino acid residues with less than a 5% molar ratio, which means that most functional amino acid sequences (such as the RGD sequence) will not be significantly affected 15. Specifically, the RGD sequence does not contain groups that will react with MA, which ensures that GelMA maintains good cell adhesion properties, which also preserves the excellent biocompatibility and bioactivity of gelatin, as well as higher solubility and lower antigenicity 13,35. More importantly, the polymerization of gelatin and MA endows GelMA hydrogel with photocrosslinking properties, which can generate methacryloyl skeleton, which can meet the needs of transplantation and treatment of different diseases by mechanically fine-tuning the generation rate, pH and light time 36. GelMA hydrogel can also induce mesenchymal stem cells (MSCs) to produce more ECM, proteoglycan, collagen II, and aminoglycan, etc., which ensures the nutrient supply required for cell growth and proliferation, thereby promoting tissue repair. In addition, GelMA hydrogel is very similar to the natural ECM, which is conducive to the adhesion, diffusion and proliferation of different types of cells in the gel scaffold 37. GelMA hydrogels have been widely used for musculoskeletal disorders treatment due to their suitable biological properties and tunable physical characteristics.

**Figure 1:**
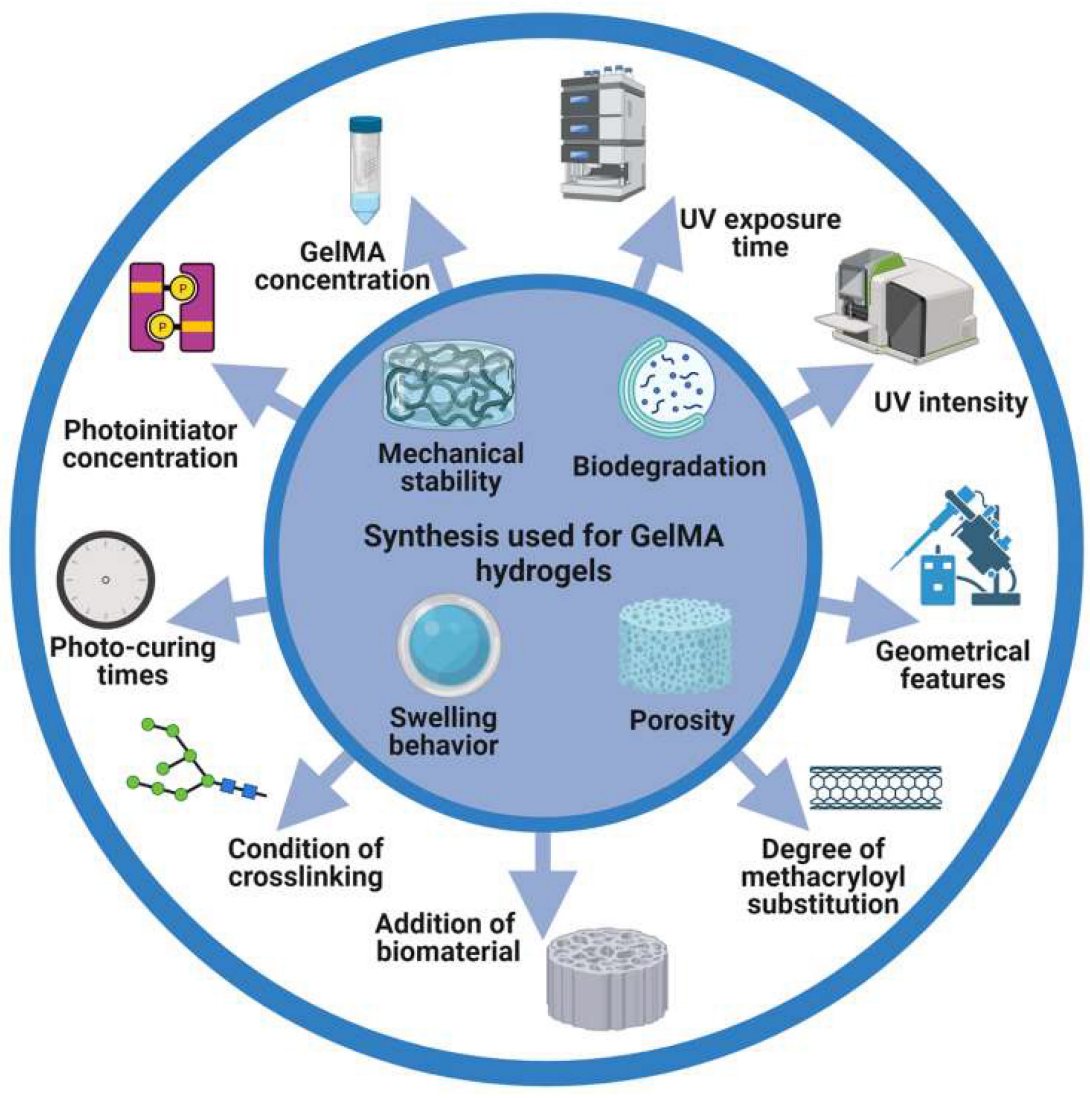
Schematic illustration of the synthesis and photocrosslinking of hydrogel in this review. GelMA hydrogel was modified to generate desired physical or chemical properties.

The introduction of photocrosslinkable methacryloyl substitution groups implies transformation of a liquid mix of a photoinitiator or cross-linkable polymer into a gelation under light irradiation. GelMA hydrogels have many adjustable mechanical and biological properties, such as mechanical properties, pore sizes, degradation rates, and swell ratio. The properties can be modified by varying the MA substitution degree, concentration (initiator and prepolymer), and photo-polymerization time (Figure 1).

**Figure 2:**
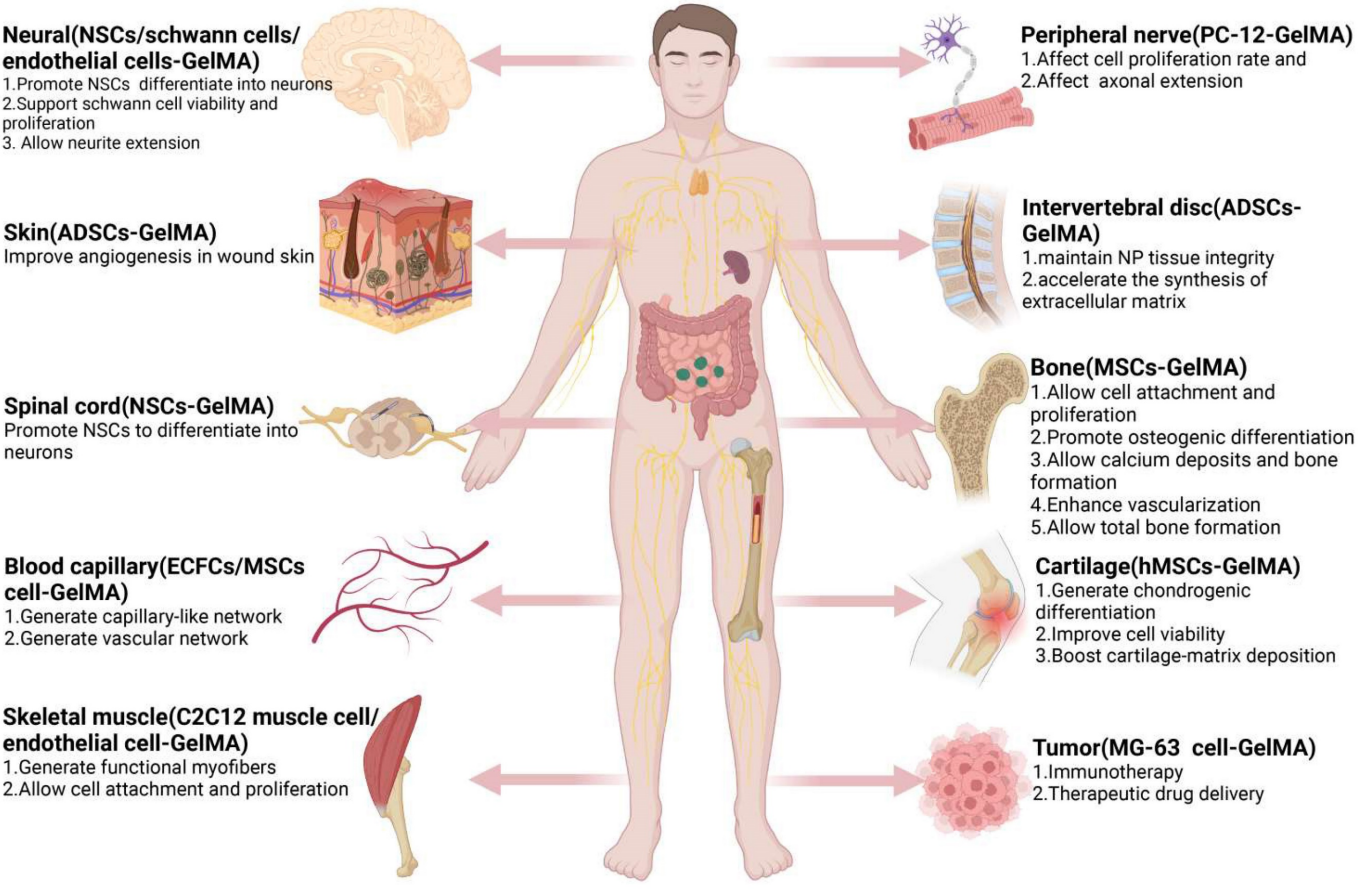
Systematic diagram illustrates the main content of this review. Applications of GelMA-based hydrogel systems for musculoskeletal disorders.

**Figure 3:**
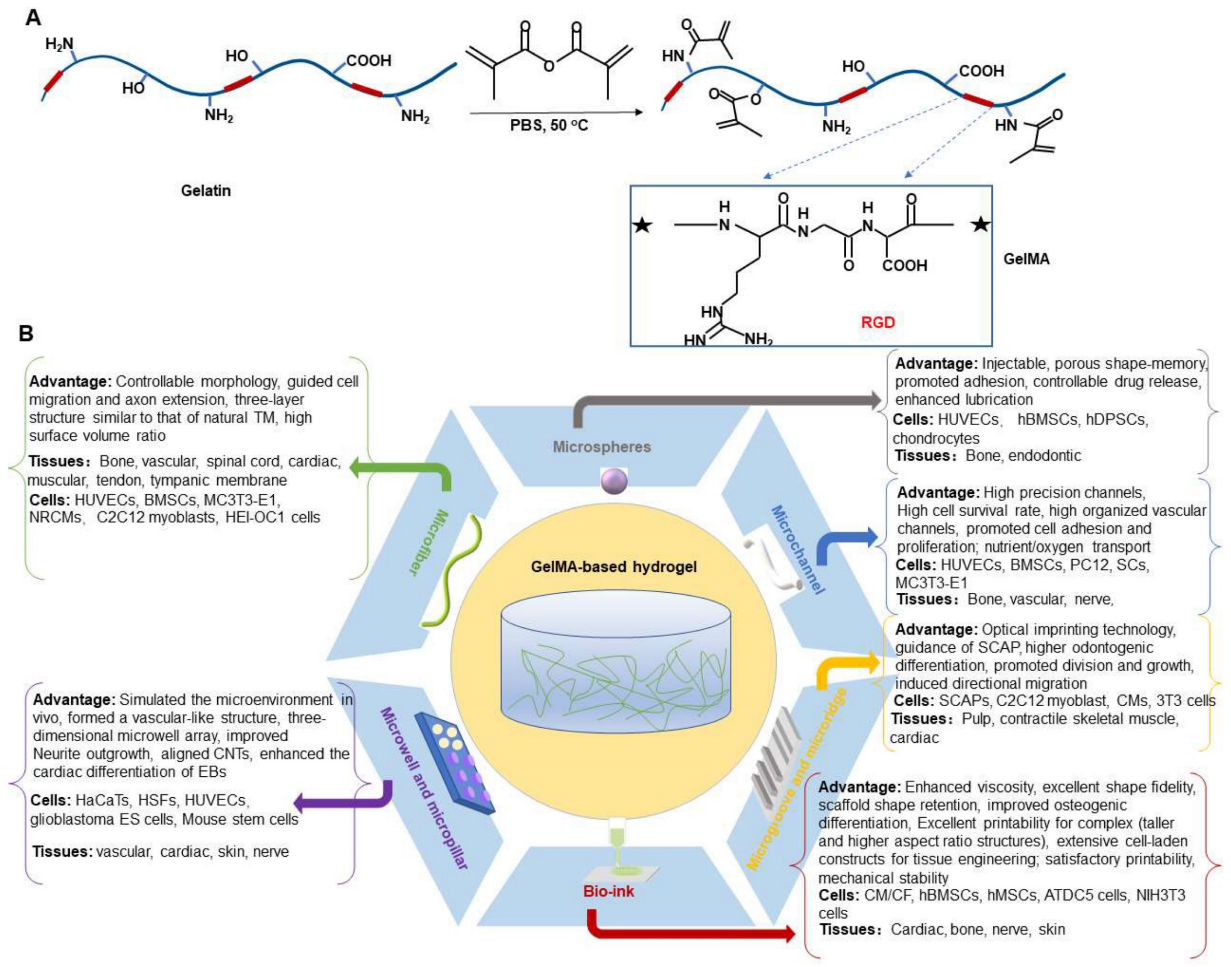
** A**) Preparation method of GelMA hydrogel. **B**) Different application forms of GelMA-based hydrogel systems for musculoskeletal disorders.

GelMA hydrogels have been used extensively in tissue engineering fields (e.g., nerve, cardiovascular, skin, bone (Table 1), and others (Figure 2). Based on different applications, GelMA hydrogels have also been prepared in different application forms to meet application needs (Figure 3). However, bone-related musculoskeletal diseases based on GelMA hydrogel have not been summarized, so this paper systematically introduces the clinical application of this disease.

## Application of GelMA hydrogel in musculoskeletal diseases GelMA for bone defect

The incidence of bone defects caused by trauma, infection, osteoporosis, metabolic diseases, tumors and deformities is increasing year by year, posing a serious threat to the physical and mental health of patients 49. For larger defects, bone tissue often has difficulty healing itself, making it a serious challenge for clinicians. Bone grafting, a common method of bone regeneration in orthopedic surgery, has been widely used to treat bone defects. Autologous bone grafting, allografting, hybrid grafting, allografting, distraction osteogenesis, induced membrane techniques and segmental prostheses are the most common surgical strategies used to treat large bone defects 50. However, the choice of the best surgical technique is still controversial, no consensus has been reached, and bone grafting is associated with risks such as infection, trauma, immunity and disease transmission. Although artificial bone has been used for disease treatment in recent years, there also remain disadvantages such as low mechanical strength, brittleness and low degradation rates 51. Compared with other bone graft substitutes, hydrogels are expected to become important materials for bone defect repair due to their good biocompatibility, porous structure like bone ECM and controllable shape 52.

**Figure 4:**
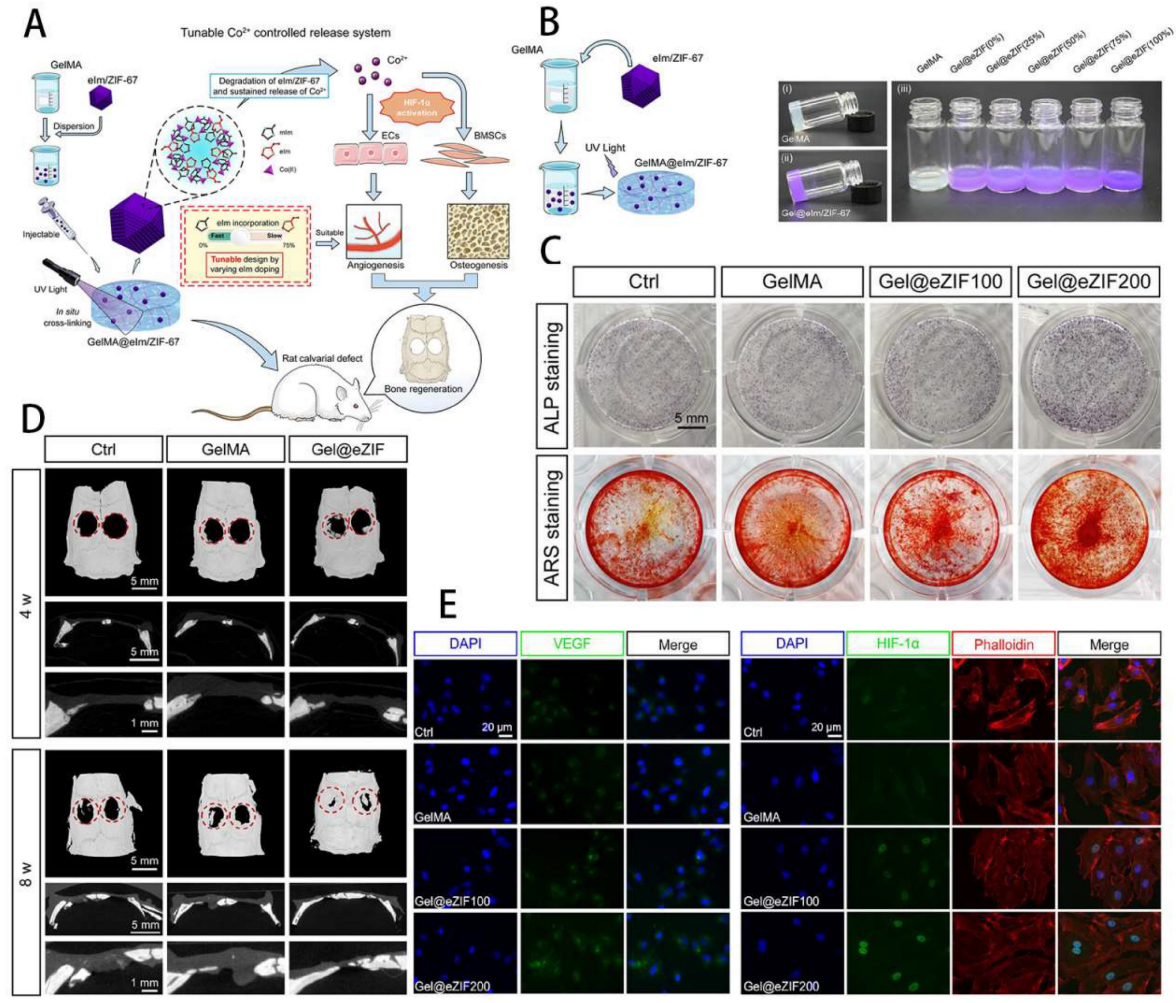
Engineering GelMA hydrogel for cartilage tissue. **A**) Schematic overview of the GelMA@eIm/ZIF-67 hydrogel for Co-ion release to enhance osteogenesis and angiogenesis through activating HIF-1α. **B**) Synthesis and digital images of the GelMA and eIm/ZIF-67-based hydrogels.** C**) Assessment of osteogenic differentiation. **D**) Micro-CT images of bone tissue. **E**) Expression level of VEGF and HIF-1α. Reproduced and adapted with permission from ref. 62. Copyright 2021 American Chemical Society.

GelMA hydrogels not only have unique photocrosslinking properties, but also have controlled mechanical properties, controlled degradation properties and good biocompatibility, making them widely used in the study of bone defects 53. It was found that GelMA not only mimics the ECM environment of bone and promotes osteogenic differentiation of stem cells, but can be combined with other materials, cells, cytokines, drugs and exosomes to enhance its ability to repair bone defects. As shown by Fang et al. 54, the Bio-GelMA hydrogel scaffold mimicked bone ECM and found that when co-cultured with adipose-derived stem cells (ADSCs), it significantly promoted the osteogenic differentiation of ADSCs. Bhattacharyya et al. 55 doped amorphous calcium phosphate micro/nanoparticles (CNP) into GelMA and fabricated CNP/GelMA polymer by 3D printing to find the best solution to promote bone regeneration by adjusting different sizes and shapes. Cao et al. 56 used 3D printing technology to manufacture PCL/TCP/GelMA porous scaffolds, encapsulating fibroblasts, BMSCs and osteoblasts in their scaffold, which showed excellent pro-cell proliferation, viability and bone and cartilage differentiation. The importance of antimicrobial resistance needs to be taken into account in the clinical treatment of infected bone defects. Qian et al. 57 constructed a composite Ta/GelMA/PLGA/Van scaffold, which not only loaded vancomycin (Van) to achieve excellent bacterial inhibition, but also had good biocompatibility and osseointegration ability. Innervation is an important initiator of bone regeneration and is involved in the process of bone growth and repair, so the importance of nerve regeneration needs to be taken into account when using tissue-engineered scaffolds to repair bone defects. In this way, Jing et al. 58 used magnesium-modified black phosphorus (BP@Mg) incorporated into GelMA to prepare a photosensitive conductive hydrogel for infected bone defects and found that it not only had efficient antibacterial properties, but also promoted nerve and bone growth. Vascularised osteogenesis is essential for successful bone regeneration, so promoting vascular growth while treating bone defects is an important strategy. BMP2 was found to enhance the coupling of angiogenesis and osteogenesis to improve bone regeneration in bone defects 59. Chen et al. 60 exploited the intrinsic angiogenic potential of vascular-derived ECM (vECM) and its specific affinity for growth factors to construct the vECM/GelMA hydrogel delivery system, a system that effectively modulates BMP-2 release and has demonstrated excellent pro-angiogenic and pro-osteogenic properties in both in vivo and in vitro experiments. Exosomes have important roles in bone repair, including promoting stem cell migration and promoting angiogenesis. Yang et al. 61 therefore designed an injectable MMP1-sensitive hydrogel microsphere mixed with a self-assembling peptide (KLDL-MMP1), GelMA and BMSC-Exos, which significantly promoted angiogenesis and bone repair. Sun et al. 62 prepared a GelMA@eIm/ZIF-67 composite hydrogel, which can regulate and control the release of Co ions, and effectively promote the osteogenic differentiation of BMSCs and angiogenic activity of human umbilical vein endothelial cells (HUVECs). The rat calvarial defect model showed that GelMA@eIm/ZIF-67 hydrogel exhibited neovascularization and new bone formation with high biocompatibility, making it ion-controlled release system and a proangiogenic/osteogenic technique of bone defects treatment (Figure 4). Some researchers have also focused on both vascular and neural regeneration when designing composite hydrogels to promote bone regeneration. For instance, Xu et al. 63 doped magnesium ion-modified black phosphorus (BP@Mg) nanosheets into GelMA hydrogels to prepare the upper hydrogel, while the bottom hydrogel was designed as a double network hydrogel system consisting of two interpenetrating polymer networks composed of GelMA, PEGDA and β-TCP nanocrystals. The upper layer was found to promote neural and vascular regeneration, while the lower layer significantly promoted osteogenic differentiation of BMSCs, providing a new idea for the clinical treatment of bone defects. The periosteum is an important tissue membrane for skeletal development and injury repair, and its importance should be emphasized in the study of bone repair. As Sun et al. 64 have used GelMA as a basis for the preparation of bio-ink into artificial periosteum through 3D printing and other techniques, both *in vivo* and *in vitro* experiments have demonstrated its excellent osteogenic effect. It is evident that GelMA has been extensively investigated in the repair of bone defects, and can not only promote stem cell osteogenesis, but also play a variety of roles in concert with various cells, extracellular vesicles, drugs and cytokines. In addition, GelMA composite hydrogels can simultaneously promote the growth of neurovascular and periosteal membranes to expand the ability to repair bone defects.

It is worth mentioning that although GelMA hydrogel has good osteogenic properties such as cell adhesion, proliferation and differentiation, the lack of mechanical properties limits its application, so it is urgent and necessary to improve the mechanical strength of GelMA hydrogel for better use in musculoskeletal diseases. By combining it with other materials, the mechanical properties of GelMA can be enhanced. Wang et al. 65 prepared a new two-component polymer hydrogel GelMA-Dex MA using GelMA and dextran glycidyl methacrylate (Dex MA) by photocrosslinking polymerisation. By controlling the degree of substitution (DS) in Dex MA, good mechanical properties can be obtained. The combination with a scaffold with excellent mechanical properties is also an important way to improve the mechanical properties of GelMA. Such as Ma et al. 66 who developed a Ti-6Al-4V alloy/GelMA hybrid scaffold with biomimetic features (GMPT) for bone defect repair and studies showed better osteogenic and angiogenic capabilities. Additionally, optimising the duration of UV exposure can improve mechanical properties while maintaining cell viability and promoting cell differentiation levels 67. It was found that the mechanical properties of GelMA hydrogels were also influenced by GelMA concentration 68, initiator concentration, photopolymerisation method and number of light cures 69, methacrylate-substitution efficiency, cross-linking conditions, geometrical characteristics 70 and other factors. Hence, when studying bone defects, it is necessary to optimise the mechanical environment of GelMA, to use it in synergy with other excellent biomaterials, cells and factors, and to construct excellent composite materials through 3D printing and other techniques is the direction of later research and a necessary part of pre-clinical research.

### GelMA for Osteoarthritis (OA)

Osteoarthritis (OA), a common musculoskeletal disorder, involves structural changes in articular cartilage, subchondral sclerosis, osteophyte formation, meniscus denaturation, synovial hyperplasia, and hypertrophy of capsular ligament. In OA, knee is the most prevalent joint affected 71. Specific medications given orally can reduce inflammation in early stages of OA. However, due to the avascular nature, articular cartilage possesses limited ability to self-repair and regeneration ability and absorption is exceedingly low 72.

Even though artificial joint replacements are still recommended for advanced OA in clinics, the surgical approach still has drawbacks including increased friction and wear 73. Therefore, developing an innovative, non-surgical technique to improve local delivery systems and the lubrication condition is essential for maintaining joint function. Furthermore, it is beneficial for treating OA inflammatory symptoms and reducing excessive cartilage friction and wear.

Hydrogel has been widely employed to produce cartilage repair materials because it has biomimetic cartilage tissue features such as high water content, flexibility, and friction resistance. GelMA has the properties of both natural and synthetic biomaterials, has a three-dimensional (3D) structure suitable for cell growth and differentiation, excellent biocompatibility, and cell-responsive properties, and can be used to replace artificial basement membranes or other natural collagen hydrogels. GelMA also exhibits good thermosensitive gel characteristics and degradability, as well as adjustable mechanical qualities.

Microcarrier applications represented by GelMA have made great strides in cartilage tissue engineering and can provide a suitable microenvironment for chondrocyte development 74. The application of GelMA in cartilage repair include three aspects, cooperate with other materials to promote cartilage regeneration, load active factors to enhance cartilage regeneration ability, and load seed cells to provide the driving force for cartilage regeneration. GelMA hydrogel itself can promote chondrocyte adhesion and proliferation, and induce the differentiation of cells into chondrocytes. Yang et al. 75 produced a functional gelatin hydrogel scaffold (GelMA-AG) chemically modified with alanyl-glutamine (AG), which can activate the energy metabolism of chondrocytes, regulate the metabolic microenvironment, reduce cartilage inflammation and ROS, resulting in effective promotion of cartilage repair.

GelMA cooperates with other materials (such as natural polymers, small molecules, metal ions, etc.) to construct multifunctional composite carriers under the action of physical or covalent cross-linking to promote cartilage regeneration, which is one of the current strategies for cartilage repair 76-79. By constructing a hydrogel scaffold with a double network cross-linking pattern of GELMA and polyethylene glycol diacrylate (PEGDA) and encapsulating KGN in a bionic scaffold (GELMA/PEGDA + KGN), Yu et al. 80 found that it could induce stem cells to homing and differentiate into chondrocytes to repair the defective cartilage tissue, and also ensure certain structural and mechanical properties.

**Figure 5:**
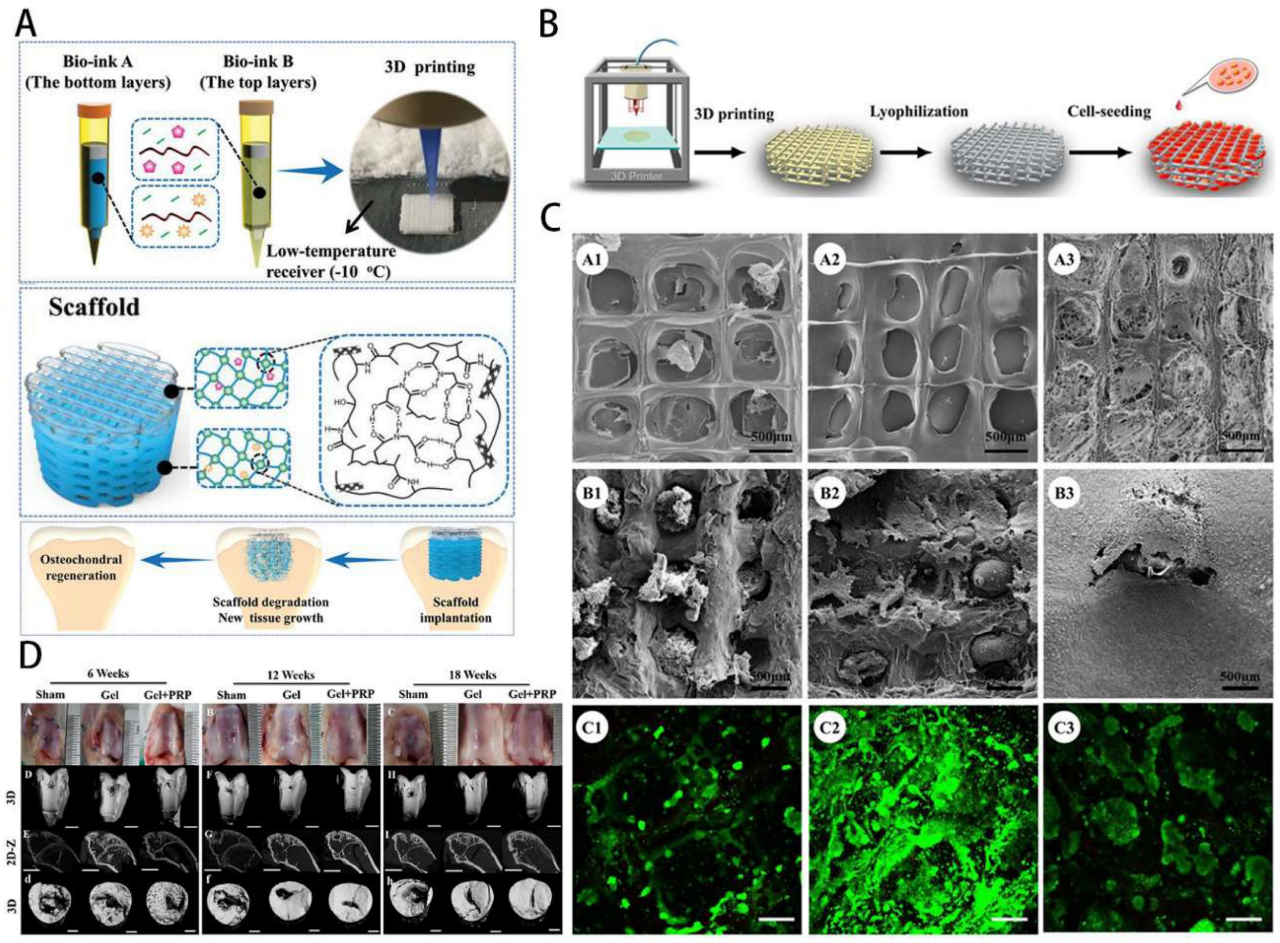
GelMA-loaded nanoplatelets in bone tissue engineering. **A**) Schematic overview of GelMA bioprinting of the scaffolds for osteochondral repair. Reproduced with permission from ref 79. Copyright 2019 Wiley. **B**) GelMA scaffold preparation by photocuring GelMA bioprinting and lyophilization. **C**) Structure properties and biocompatibility of GelMA scaffolds. Reproduced with permission from ref. 76. Copyright 2018 American Chemical Society. **D**) Gross images and Micro-CT observation of osteochondral repair by scaffolds. Reproduced with permission from 84. Copyright 2021 Elsevier.

A single GelMA hydrogel scaffold is difficult to achieve efficient and rapid healing of severe cartilage defects, so it is necessary to load active factors to enhance the regeneration ability of cartilage. GelMA hydrogels in cartilage repair field commonly used as a carrier, load outside the bioactive factors such as bleeding, extracellular vesicles and cartilage extracellular matrix, drug molecules such as diclofenac sodium and triamcinolone acetonide in cartilage defect parts gradually slow-release achieve anti-inflammatory, stimulate the purpose of the cartilage cell proliferation differentiation, in order to promote cartilage repair 81-84 (Figure 5D). To attain favorable anti-inflammatory and cartilage regeneration, Sun et al. 85 prepared a curcumin delivery system based on PEG-GelMA [poly (ethylene glycol) dimethacrylate-gelatin methacrylate] hydrogel microgels. Through in vivo and in vitro experiments, it was demonstrated that PGMs could attenuate the inflammatory response of chondrocytes stimulated by IL-1β, effectively protect cartilage tissue and induce cartilage regeneration.

GelMA hydrogel has ECM-like characteristics and can provide an environment and components for cell growth. It is often used as a seed cell delivery carrier to load chondrocytes, BMSCs, fibroblasts and other seed cells to promote the repair of cartilage through the proliferation, migration and differentiation of seed cells 86,87. Gao et al. 79 successfully constructed biodegradable high-strength poly(N-acryloyl-2-glycine) (PACG) by biohybrid gradient hydrogel to repair osteochondral defects. Relevant research showed that the biohybrid gradient hydrogel supported the adhesion and spreading of loaded cells and enhanced the chondrogenic and osteogenic differentiation-related gene expression of BMSCs. Implantation experiments showed that biohybrid gradient hydrogel scaffold could significantly regenerate the articular cartilage and subchondral bone (Figure 5A). Xia et al. 76 successfully prepared adult ear-nose-shaped scaffolds by integrating photo-curing 3D printing and freeze-drying techniques by mixing gelatin and hyaluronic acid, which exhibited enhanced mechanical strength and slowed down to match the cartilage regeneration degradation rate. In addition, the interaction of the chondrocytes with the scaffolds successfully regenerated mature cartilage with typical caveolae structure and cartilage-specific extracellular matrix in autologous goat models (Figure 5B-C). Recent research has focused on the construction of composite hydrogel systems based on GelMA. By adding specific factors or drugs, together with cellular therapy, they mostly provide the growth environment required by chondrocytes and actively stimulate cartilage development and reduce the inflammatory response, which can ultimately treat osteoarthritis.

### GelMA for intervertebral disc degeneration (IDD)

Low back pain (LBP) is a group of the lower back, lumbosacral and hip pain for the main symptom. The incidence of lumbago is increasing year by year and has become a serious problem affecting human health. IDD is the leading cause of LBP and disability, particularly in the older adults. As an age-related disease, the pathogenesis of IDD is still not completely clear; inflammatory response and fine apoptosis may be the main cause of IDD 88. When symptoms related to endplate inflammation and spinal cord compression occur. Conservative treatment is often ineffective, and most patients will eventually choose surgical treatment, such as nucleus pulposus excision, lumbar interbody fusion, and so on 89. Surgery can cause inflammation and bone fusion and other complications; these complications have been the difficulty clinicians face the topic. Therefore, it has been necessary to develop novel therapeutic options for IDD. Hydrogels are viewed as an effective alternative strategy to treat this disease due to their good physicochemical tunability 90. Ideal IVD repair hydrogel material for regenerating IVD should possess the following features: (1) excellent biocompatibility and biodegradability; (2) injectability for conservative management or minimally invasive intervention; (3) in situ curing to avoid leakage; (4) strong biomechanical characteristics; (5) good drug-loading and release performance; (6) successful integration and adherence with target to prevent poor localization 91.

**Figure 6:**
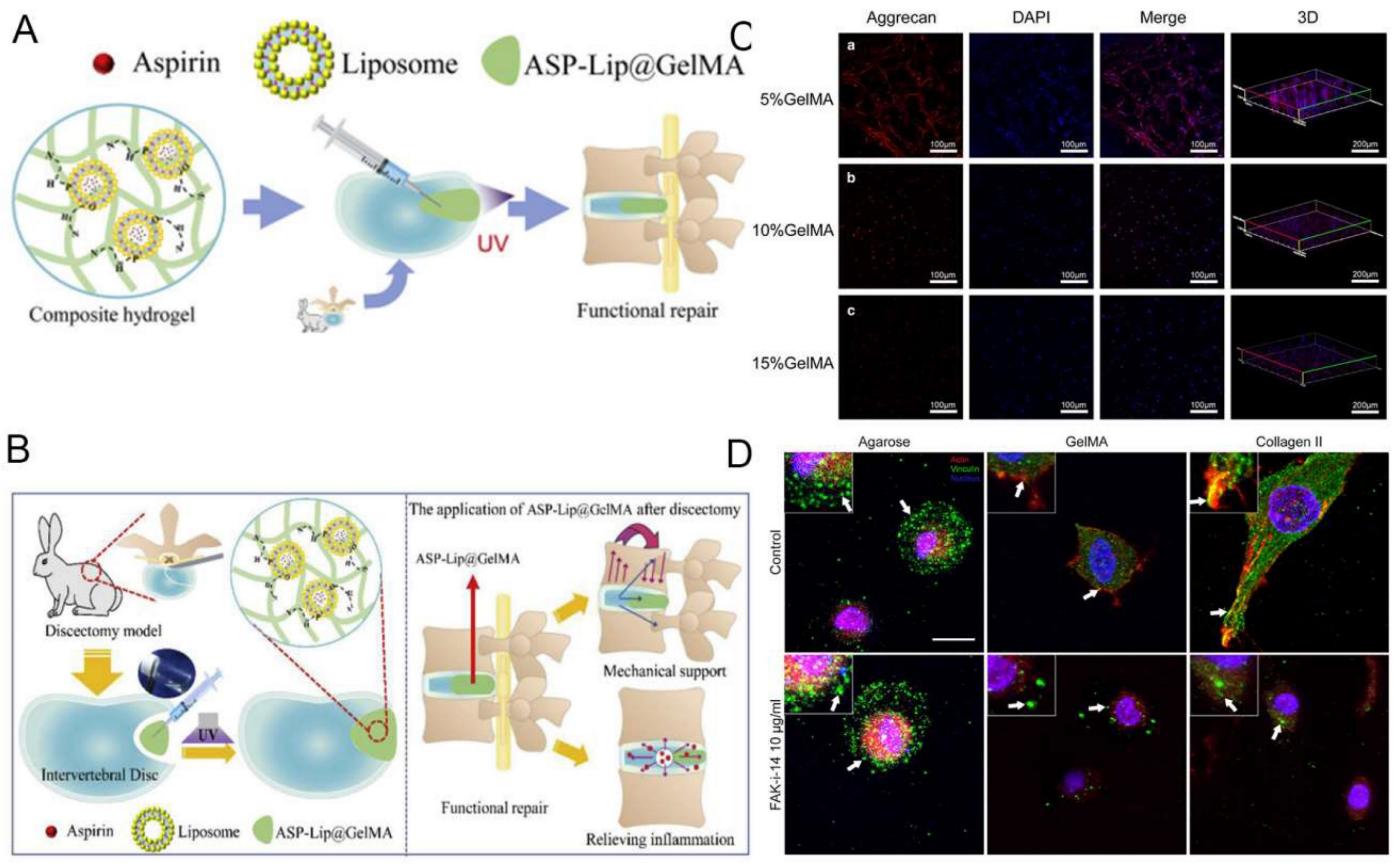
GelMA-based hydrogel for intervertebral disc engineering. **A**) Schematic overview of GelMA scaffolds for intervertebral disc degeneration.** B**) Expression of Col II of NPCs-loaded hydrogel. Reproduced and adapted with permission from ref 95. Copyright 2021 Elsevier. **C**) GelMA hydrogel for preventing recurrence after partial discectomy. Reproduced and adapted with permission from ref 93. Copyright 2020 Wiley. **D**) Expression level of aggrecan of NPCs-loaded hydrogel. Reproduced and adapted with permission from ref 97. Copyright 2018 Elsevier.

GelMA hydrogel is a photosensitive biological hydrogel material, with good biocompatibility, mechanical properties adjustable, light curing properties and biological activity. As a novel tissue engineering material, GelMA hydrogel has become Hotspot of biomedical application research, especially in the field of orthopedics. GelMA hydrogel dries fine by carrying cells, growth factors, drugs, promote bone fusion, eliminate IVD inflammation disease response and promote nucleus pulposus (NP) tissue regeneration 92. It lays a biological foundation for surgical treatment of intervertebral disc degeneration (IVDD) (Figure 6A). Xu et al. 93 demonstrated that the 5% GelMA hydrogel showed the optimal performance in morphology and proliferation of cells, as well as the aggrecan and Col II expression (Figure 6C). Increasing evidence indicates that inflammation may show a further increased in IDD than healthy IVD in the progression of spinal degeneration 94.

GelMA hydrogel can load anti-inflammatory drugs and delivers them precisely to the site of inflammation. Liu et al. 95 synthesized a crosslinked ASP-Lip@GelMA hydrogel with anti-inflammatory properties. Full inflammatory cycle coverage should be achievable by inhibiting inflammatory factor expression, thereby restoring mechanical stability and preventing IDD recurrence (Figure 6B).

NP degeneration is the major cause of degenerative disc disease (DDD). GelMA hydrogel with high water content and fluid pressure characteristics can facilitate the infiltration of oxygen and nutrients, providing a favorable microenvironment for NP cells proliferation and differentiation 96. Anita et al. 97 compared the formation of glycosaminoglycan (GAG) deposition and focal adhesion of NP cells in hydrogels. They prove that focal adhesion signals are involved in the reaction of NP cells in hydrogels containing integrin binding sites (that is, GelMA and type II collagen) (Figure 6D). Li et al. 98 inoculated NP cells and annulus fibrosus-derived stem cells (AFSC) in different concentrations of GelMA hydrogel scaffolds and found that GelMA provided a suitable environment for cell survival and that the different mechanical properties significantly affected cell adhesion as well as the major components of the ECM. This approach may provide a new approach to the regeneration of IVD. Imbalance of oxygen metabolism is an important trigger of IVD inflammation. Clinically, specific hydrogels can be constructed by combining antioxidants with GelMA, which can reduce the inflammatory response and degradation of ECM. For instance, Li et al. 99 constructed engineered hydrogel microspheres for oxygen metabolism homeostasis (GM@CS-BP) by grafting chitosan nanoparticles encapsulated with strongly reducing black phosphorus quantum dots (BPQDs) onto GelMA microspheres via amide bonds, and demonstrated through in vivo and in vitro experiments that this microsphere can regulate oxygen metabolism, reduce inflammation and promote NP regeneration. It can be seen that GelMA hydrogel grafts have the potential to be an effective tool in the field of tissue engineering for the treatment of IVDD.

### GelMA for tendon disorders

Tendon disorders, common musculoskeletal conditions, account for 30% of general practice consultations for musculoskeletal consultations, resulting in significant disability and pain 100.

Tendon disorders are subject to high tensile loads and are easily torn as a result of a variety of factors, including genetic factors, chronic trauma, inflammation, and aging 101. Non-operative management, such as nonsteroidal anti-inflammatory medications, ultrasonography, and physiotherapy, is considered to be the first-line treatment. The rehabilitation times from the tendon pathologies might be extensive and the outcomes are often unsatisfactory 102. The rehabilitation has been hindered by the lack of insight into the mechanisms associated with pathogenesis of tendon disorders. This is partly owing to the innate low cellular and hypo-vascular nature of tendon renders it lacking sufficient healing ability 103.

Furthermore, lack of a clear picture of the cellular and molecular mechanisms that cause tendonopathy makes developing novel and effective therapeutic strategies difficult 104. Thus, tendon regeneration in musculoskeletal illnesses has long been a difficult topic to solve.

There are still many debates over the role of inflammation in tendinopathy development 105. Recent studies suggest that a cascade of inflammatory processes, including inflammatory mediator production, imacrophages and lymphocytes infiltration, and MMPs activation, play a critical role in the etiology of tendinopathies 106,107. Interleukin-1β, among other cytokines, can increase tendinopathy by activating MMPs, which leads to catabolic destruction of ECM. As a result, minimizing aberrant ECM remodeling and addressing the inflammatory cascade is a good way to treat tendinopathy.

**Figure 7:**
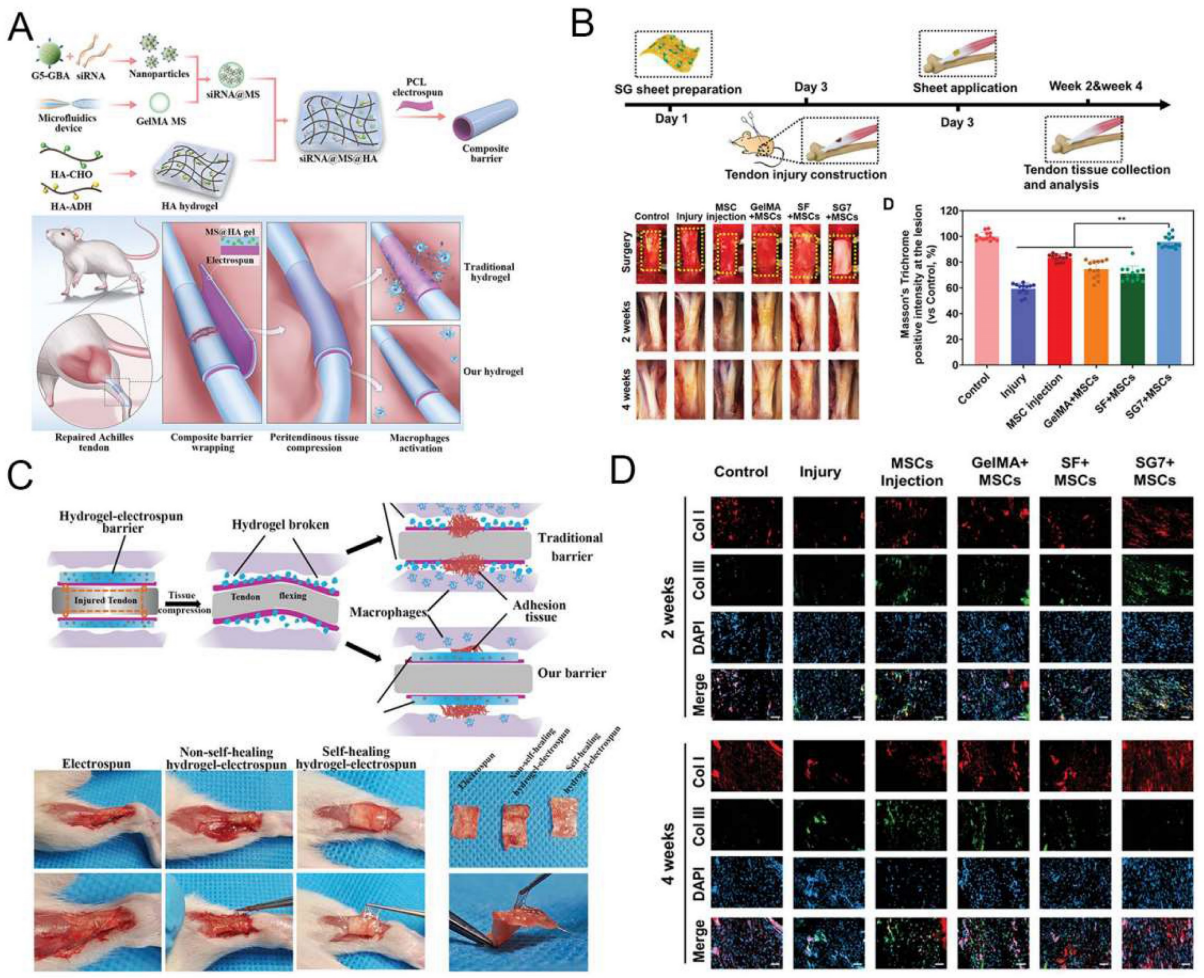
Implantation of GelMA-based hydrogel for tendon disorders.** A**) The fabrication process of the siRNA@MS@HA hydrogel. Reproduced and adapted with permission from ref 112. Copyright 2022 Wiley. **B**) Timeline and diagram of achilles tendon repair. Reproduced and adapted with permission from ref 113. Copyright 2022 Wiley. **C**) Schematic diagram of macroscopic images in the antiadhesion barrier in peritendinous tissue. Reproduced and adapted with permission from ref 112. Copyright 2022 Wiley.** D**) Immunofluorescence images of expression level of tendon tissues. Reproduced and adapted with permission from ref 112. Copyright 2022 Wiley.

Previous studies indicated that in situ injection of a formable biodegradable hydrogel was effectively heal tendon injuries while minimizing patient suffering 108-110. Because of the unique physiological structure of tendons, injectable hydrogel is a better choice than other biological materials. Much research has lately looked into the viability of using GelMA hydrogel to treat tendon diseases. Due to the inclusion of cell attachment and matrix metalloproteinase-reactive peptide motifs, GelMA hydrogels are similar to several basic features of natural ECM, allowing cells to proliferate and spread in GelMA-based scaffolds 19. GelMA can also be combined with nanoparticles and other polymers to create a hybrid hydrogel with biological applications 111. Cai et al. 112 proposed that MMP-2-degradable GelMA microspheres (MSs) encapsulated with Smad3-siRNA nanoparticles are entrapped within the HA hydrogel to prevent peritendinous adhesion and inhibit fibroblast proliferation. MSs degrade responsively to ensure on-demand release of siRNA nanoparticles, which are highly effective in reducing inflammation and inhibiting tendon adhesions (Figure 7A and 7C). Xue et al. 113 combined GelMA with SF to create a nanofiber scaffold (SG) with high biological activity and mechanical strength. Compared with SF nanofibers, mesenchymal stem cells (MSCs) seeded on SG fibers with the best composition (SG7) showed enhanced growth, proliferation, vascular endothelial growth factor production, and tendon gene expression. MSCs conditioned medium cultured on SG7 scaffold can greatly promote the migration and proliferation of tendon cells. Histological analysis also showed that the SG7 scaffold enhanced tendon tissue regeneration in vivo (Figure 7B and 7D). Rothrauff et al. 114 has used ADSCs-loaded GelMA hydrogel to repair chronic tendon injuries. After bone marrow stimulation, kartogenin (KGN)-loaded hydrogel scaffold improves tendon enthesis healing and promote mechanical property of fibrous cartilage. In addition, Huang et al. 115 constructed a GelMA hydrogel scaffold loaded with KGN and demonstrated that bone marrow stimulation of this scaffold could repair tendon injuries by improving mechanical properties and promoting chondrogenesis through a *in vivo* New Zealand rabbit model. Thus, GelMA-based hydrogels can provide a suitable living environment for the cells and promote the differentiation of stem cells into tendons, making them a promising treatment for the repair of tendon injuries.

### GelMA for skeletal muscle disease

Skeletal muscles, which are made up of muscular fibers bound together by epimysium and whose functional capacities are strongly correlated with their well-organized microstructure, are critical in the body's diverse posture and sports maintenance. The body is made up of over 600 skeletal muscles that account for about 40% of the total weight. Muscle dystrophies and other degenerative muscle illnesses induce massive muscle cell loss. Scar tissue formation impeded the endogenous regenerating potential in cases of substantial muscle loss, compromising the native muscle structure and finally resulting in significant dysfunction over time. A substantial amount of tissue must be replaced after volumetric muscle loss in order for function to be restored 116. Skeletal muscle tissue engineering is devoted to developing bioartificial muscle tissue constructs to accelerate the process of regeneration, has been shown to be a very promising strategy for skeletal muscle injury repair.** Table 2** summarizes the advances in development and applications.

**Figure 8:**
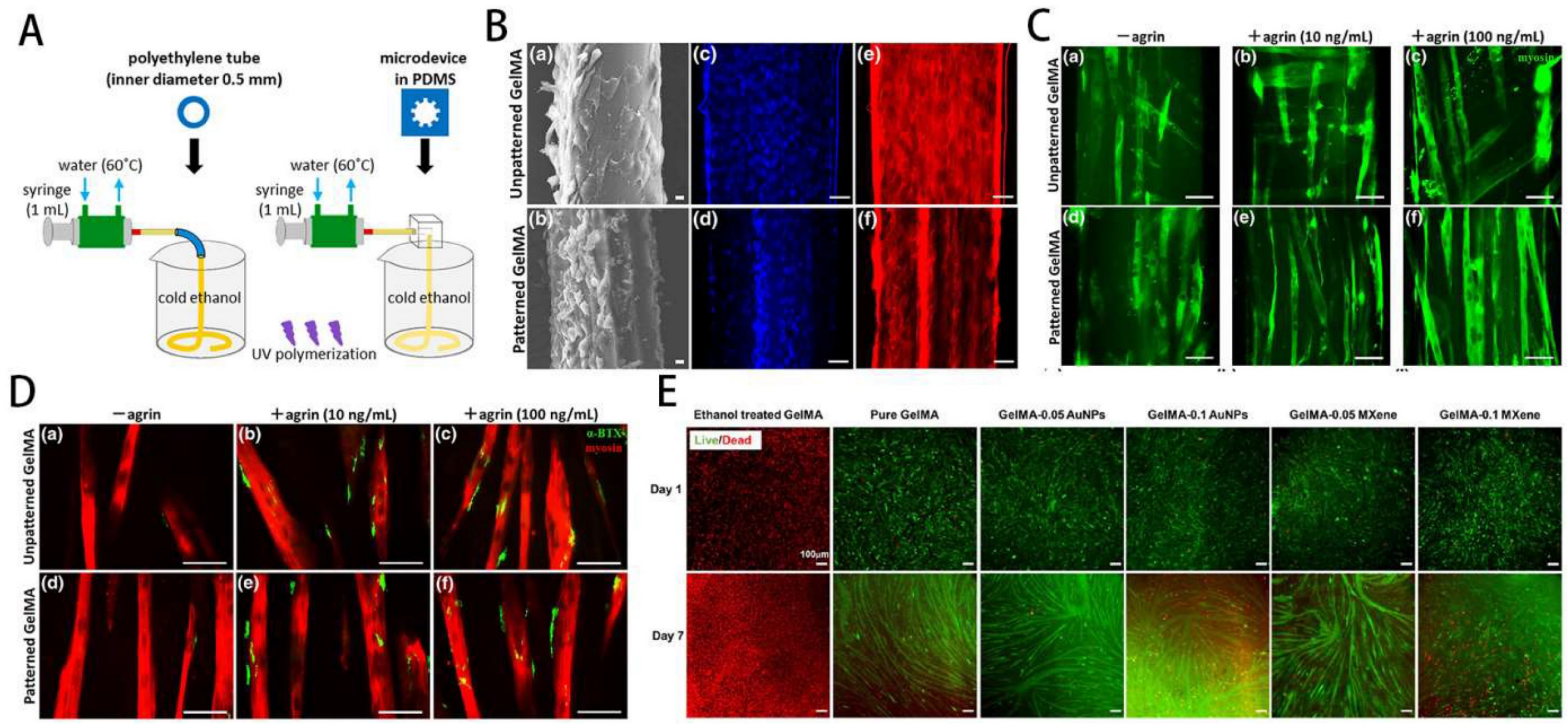
GelMA-loaded hydrogel for skeletal muscle regeneration. **A**) Schematic overview of fabrication of micropatterned and unpatterned GelMA fibers. **B**) FE-SEM photographs show myoblasts on GelMA fibers. **C**) Fluorescent photographs of myotubes on GelMA fibers. Reproduced and adapted with permission from ref 118. Copyright 2018 Wiley.** D**) Fluorescent photographs of AChR clusters on myotubes. **E**) Biocompatibility and conductivity of GelMA-based MXene and AuNPs hydrogels. Reproduced and adapted with permission from and ref 125. Copyright 2021 American Chemical Society.

GelMA has been extensively utilized as an extracellular matrix (ECM) mimicking scaffold for tissue engineering applications. However, hydrogels are generally non-conductive, which limits their ability to modulate the function of certain cells sensitive to electrical stimulation, such as nerves and muscle cells. Hydrogels with conductive properties, as biologically active scaffolds, have the effect of electrical stimulation and functional adjustment of cells and have been greatly developed in recent years. In skeletal muscle tissue engineering, seed cells and scaffold materials are often used for tissue repair. Myoblasts, most widely employed seed cells for muscle tissue engineering, have a strong ability to differentiate and proliferate. Selwa et al. 125 Nanocomposite conductive bioinks based on low-concentration MXene nanosheets/gold nanoparticles and GelMA can promote C2C12 cell proliferation, differentiation, and gain 3D printability of skeletal muscle tissue. The cells-loaded GelMA fiber was exposed to electrical stimulation, which can facilitate the growth of muscle fibers (Figure 8E).

Yang et al. 126 reported that the C2C12-laden GelMA structure showed effective formation/maturation of functional myotube with ability to respond *to* stimulation with electric field. Using a nanoengineered, growth factor-eluting bioink, Jacob et al. 121 successfully developed C2C12 myoblasts-laden GelMA scaffold uniaxially aligned via an in-situ crosslinking process during extrusion for volumetric muscle regeneration. Ebrahimi et al. 118 fabricated photocrosslinkable GelMA fibers with surface micropatterning using microfluidic spinning. The micropattern can promote the ordered arrangement of C2C12 myoblasts and enhance the formation of myotubes during differentiation, effectively improving the function of engineered muscle tissue (Figure 8A-D).

In order to further improve the regenerative effect of tissue engineering on skeletal muscle, optimising the scaffold structure, exploring the best way to make the scaffold and optimising the mechanical properties have become a hot research area. In order to further improve the regenerative effect of tissue engineering on skeletal muscle, optimising the scaffold structure, exploring the best way to make the scaffold and optimising the mechanical properties have become a hot research area. Quint et al. 127 produced GelMA nano-functionalised scaffolds that significantly improved skeletal muscle cell proliferation and differentiation by promoting insulin-like growth factor 1 (IGF-1) release through electrostatic interactions with LAPONITE® nanoclays (NCs). To explore the best method of scaffold fabrication, Hwangbo et al. 128 used a in situ cross-linking (ISC) strategy to fabricate GelMa hydrogel scaffolds containing cells, and by using C2C12 and human adipose stem cells (HADSCs), it was clear that the improved bioprinting method could significantly improve the regenerative function of skeletal muscle. Furthermore, Li et al. 129 have combined GelMA and fibrinogen to produce interpenetrating network (IPN) hydrogels with adjustable stress relaxation by 3D bioprinting. This hydrogel not only provides a suitable microenvironment for the cells, but also significantly promotes cell proliferation and differentiation. Overall, GelMA-based 3D printed composite scaffolds show bright promise for skeletal muscle recovery and regeneration, and deserve further exploration and translation.

### GelMA for peripheral nerve injury (PNI)

**Figure 9:**
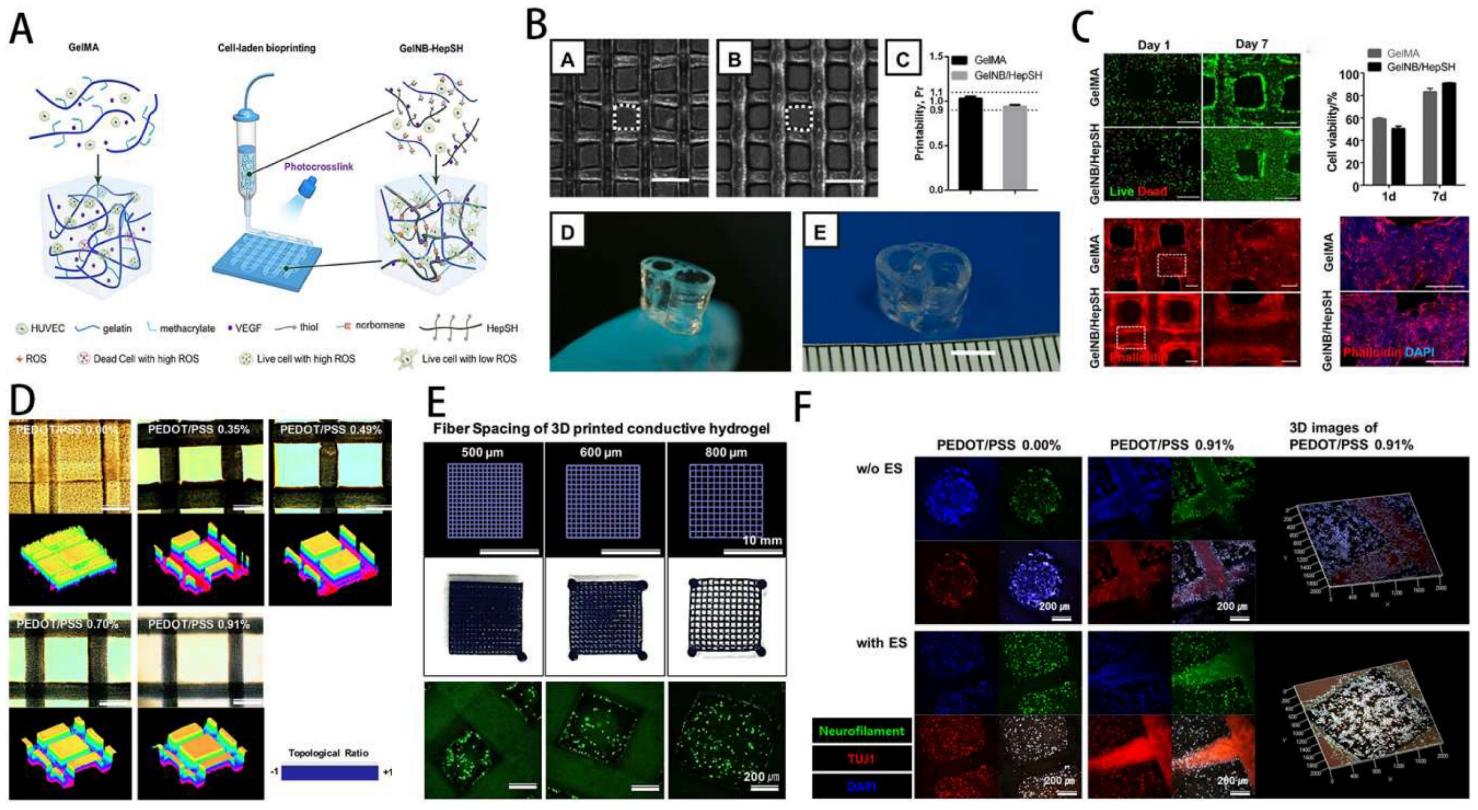
GelMA-DA hydrogel in neural tissue engineering. **A**) Illustration of cell-laden bioprinting by GelMA-based bioinks. **B**) Printability evaluation using GelMA-based bioinks. **C**) Cell-laden bioprinting by GelMA-based bioink. **D**) Optical and surface plotting photographs of photo-patterned hydrogels. **E**) Inputting AutoCAD patterns and the patterns by photocurable GelMA-based hydrogels. **F**) Immunofluorescence photographs of DRG cells-loaded GelMA hydrogel. Reproduced and adapted with permission from ref 132. Copyright 2019 Elsevier.

Peripheral nerve injury (PNI) has been among the most challenging clinical problems caused by surgery or trauma, leading to severe and permanent sensory, motor, and functional disorders of the affected innervated area. Damage to the nerves may cause nerve defects, breakage of internal blood vessels, and interruption of the interaction between neurons and tissues. Following nerve damage, a cascade of responses involving Schwann cells, macrophages, endothelial cells, and fibroblasts occurs, with lysing factors and disintegrating substances controlling most of the processes 130. The recovery of PNI requires recreation of neural circuitry and reconstruction of functional nerves. Unfortunately, even with optimal surgical treatment, functional reconstruction is rarely accomplished, and common complications include persistent pain, sensory abnormalities, and muscle paralysis. This not only poses a psychological barrier for patients, but also a more significant burden to society. Nevertheless, with severe peripheral nerve injuries, nerves' intrinsic regenerative capacity is so low that proximal axon regrowth and reinnervation are difficult to achieve. In clinical practice, the gold-standard treatment for severe nerve abnormalities is autologous nerve transplantation. However, autograft transplantation procedures for significant nerve gaps may display side effects for certain limitations, such as a lack of sources, the creation of neuromas, and donor site infection. As a result, it's critical to look for suitable alternatives to improve clinical care as soon as possible. The interval time between initial nerve damage and end-organ replantation has consistently been shown to be a key predictor of functional restoration after PNI, with proximal injuries and delayed repairs resulting in worse results. This is primarily a result of muscle atrophy and muscle denervation following denervation. Axons have to regenerate over vast distances at slow speed of 1-3 mm/day to reach distal tissues and reinnervate motor endplates following surgical repair. Loss of neuromuscular junctions (NMJs) and myofibrils in an irreversible manner lead to progressive muscle atrophy. To avoid denervated atrophy, various alternative strategies are being pursued to enhance peripheral nerve regeneration and remodel precise synaptic connections with target organs. For example, Hu et al. 131 proposed a conductive topological scaffold for neural repair by encapsulating brain-derived neurons with reduced graphene oxide (rGO) nanosheets and gelatin methacrylate (GelMA) hydrogels. Benefiting from electrical conductivity of rGO and the parallel nanoridge structure of the wing scales, PC12 cells and neural stem cells on the modified wing increased neurite length and guided cell orientation (Figure 9).

Hydrogels have ECM-like structures, which can be used for cell growth, reproduction and differentiation, and have been widely used to construct neural repair systems. Among them, GelMA is a double bond modified gelatin, which can be cross-linked and cured into gel. GelMA involves the combination of natural or synthetic biomaterials with a 3D structure suitable for cell growth and differentiation, excellent biocompatibility and cell response properties, and can replace artificial basement membranes or other natural collagen hydrogels. In addition, GelMA has good thermosensitive gel properties, degradability, and adjustable mechanical properties. It can be engineered to load with stem cells and triggers stem cells to differentiate into neurons. The application of GelMA in the field of nerve regeneration, as well as its benefits and drawbacks, are summarized as follows (**Table 3**).

The application of GelMA in nerve repair includes three aspects: synergize with other materials to promote nerve repair and regeneration, loading active factors to stimulate nerve repair and regeneration and loading seed cells to provide the driving force for nerve regeneration. GelMA hydrogel itself is suitable for cell growth and adhesion, can stimulate stem cell differentiation, and has high biosafety. GelMA is functionalized with some biologically active materials, and the optimal raw material ratio and structure are adjusted. After photocrosslinking, a nerve repair scaffold is made, which can be applied to the injured site to promote nerve repair and regeneration. Usually, the method has few kinds of raw materials, simple synthesis steps, and easy clinical transformation and application 9,131,143,144. As an example, Cai et al. 145 prepared a GelMA/SF/graphene nerve conduit and combined it with Netrin-1, a hydrogel with good mechanical properties, biocompatibility and sustainable nerve growth factor delivery for significant peripheral nerve repair and inhibition of muscle atrophy. Exosomes, cytokines, drugs, etc. are often used to regulate the microenvironment of nerve tissue damage, such as reducing oxidative stress, inhibiting inflammatory response, promoting specific cell proliferation and differentiation, etc., thereby accelerating nerve injury repair. GelMA hydrogels can be loaded with active factors or drugs to improve the immune microenvironment and stimulate nerve repair and regeneration 141,146-149. Zhang et al. 150 prepared an rGO-GelMA-PCL nerve conduit system by combining reduced graphene oxide (rGO) with GelMA and polycaprolactone (PCL), which was loaded with extracellular vesicles of bone marrow mesenchymal stem cells (BMSCs) and found to significantly repair peripheral nerves and promote vascular regeneration at the site of injury.

Stem cells have great differentiation potential and are often used as inducers to repair various tissue damages. In nerve repair, neural stem cells, bone marrow mesenchymal stem cells, dental pulp stem cells, etc. are the most commonly used seed cells, which can be loaded into GelMA hydrogel and differentiated under stimulation induction to provide the driving force for nerve regeneration 135,151-153. By studying the effect of different stiffnesses of GelMA on stem cells, Gao et al. 154 found that when the stiffness reached 2.9 kPa, it was the ideal mechanical strength for stem cells to differentiate into Schwann cell-like cells (SCLCs), and that GelMA nerve-guided catheters prepared by 3D printing technology were important candidates for promoting peripheral nerve regeneration.

**Figure 10:**
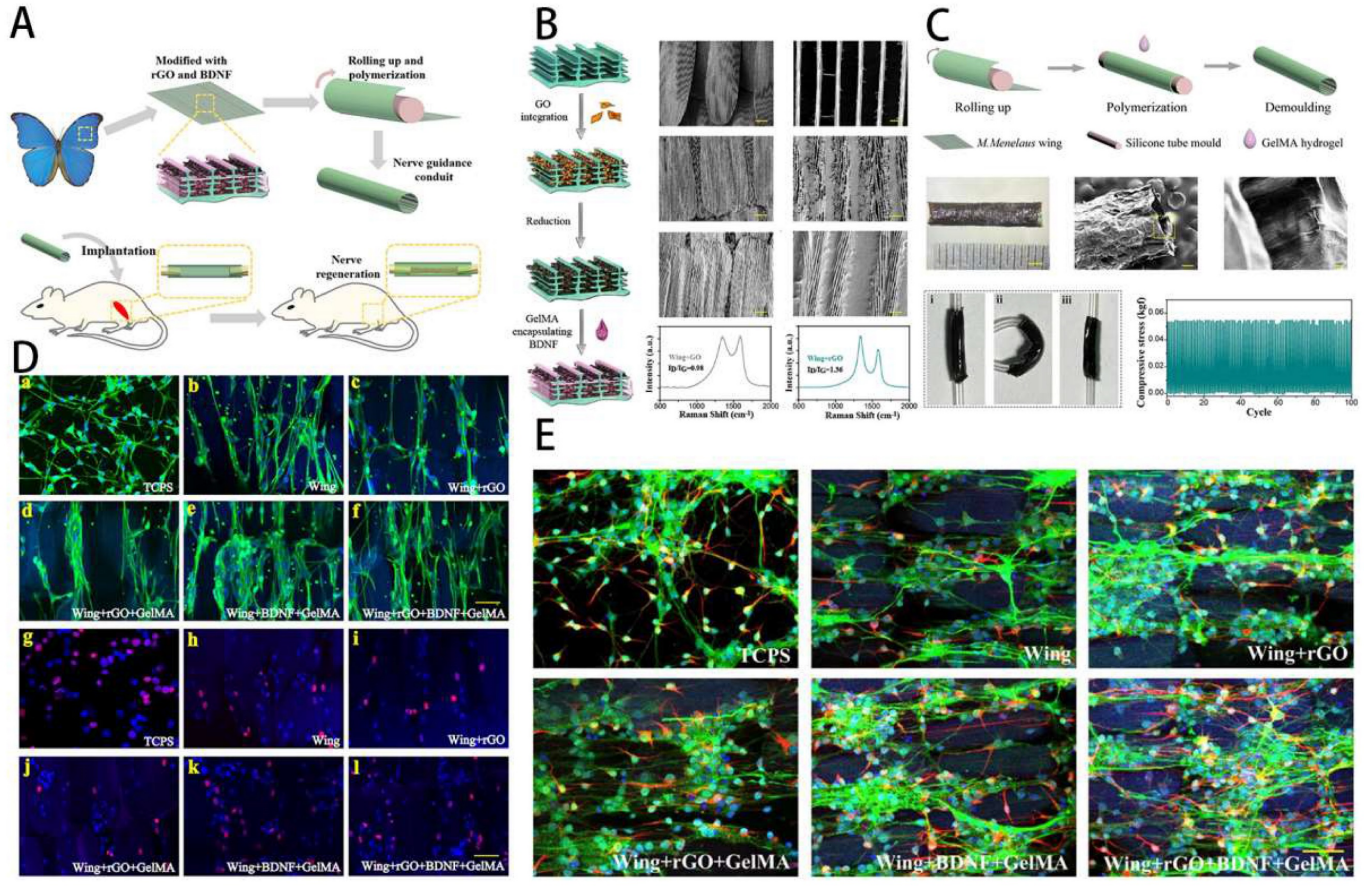
Schematic overview of the conductive NGC in peripheral nerve. **A**) Schematic illustration of NGC.** B**) Modification and characterization of the rGO/BDNF/GelMA. **C**) Construction and characterization of the NGCs. **D**) Influence of rGO/BDNF/GelMA on NSCs growth and proliferation.** E**) Influence of rGO/BDNF/GelMA on NSC differentiation and neurite extension. Reproduced and adapted with permission from ref 131. Copyright 2022 American Chemical Society.

GelMA-based scaffolds facilitate communication of electrical signals between adjacent cells, mediating the adhesion, migration, proliferation, and differentiation. GelMA hydrogels transfer electric signal to electroactive tissues and advance tissue repair through the activation of bioelectrical signaling in a tissue damage model. Therefore, more and more conductive GelMA hydrogels have been developed for nerve repair. Heo et al. 131 prepared dorsal root ganglion (DRG) cells encapsulated in GelMA hydrogel with 3D-printed conductive structures. The results proved that electrical stimulation transferred to encapsulated DRG cells could enhance neuronal differentiation (Figure 10).

### GelMA for bone tumors

**Figure 11:**
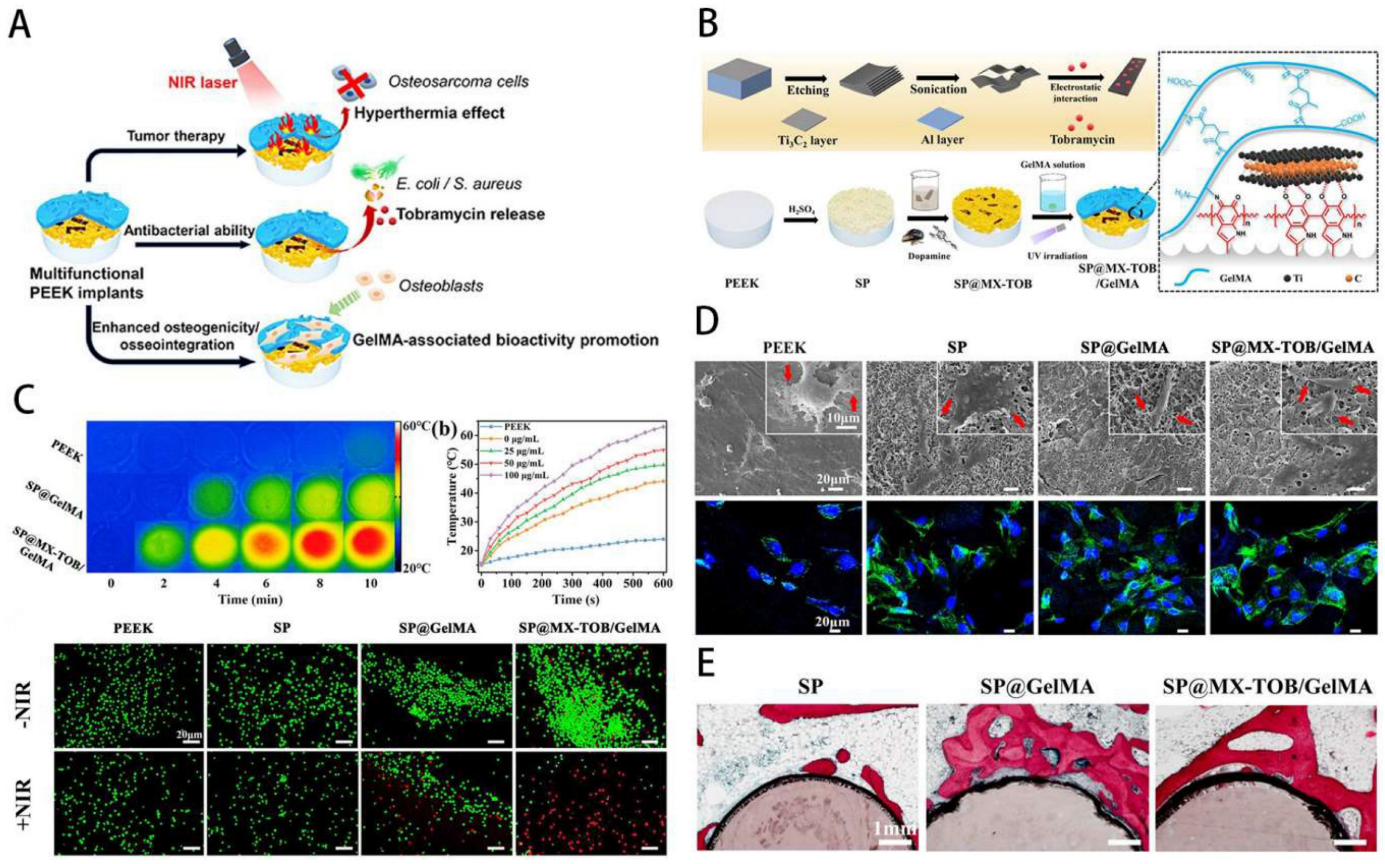
MXene-based GelMA hydrogel with osteogenicity for osteosarcoma. **A**) Schematic overview of multifunctional implants after osteosarcoma excision. **B**) Illustration of process for preparation of GelMA-based multifunctional substrate. **C**) Infrared thermal images of GelMA-based hydrogel. **D**) SEM images of osteoblasts cultured on multifunctional substrates.** E**) Histological assessment of bone contact. Reproduced and adapted with permission from ref 162. Copyright 2020 American Chemical Society.

Bone tumors can be divided into primary and secondary bone tumors. The predominant primary bone sarcomas mainly include chondrosarcomas and osteosarcomas. Tumors that advanced cancers metastasizes to the bones are called secondary bone tumors 155. Bone tumors possess a high tendency to disseminate to develop metastasis, characterized by leaky vasculatures, excessive growth, and hypoxic and acidic environment 156. Currently, stimuli-responsive biomaterials have been exploited aiming at control of target tumor tissues and release drugs in response to the stimuli 157. Studies have shown that GelMA is beneficial in the treatment of meniscus damage 158,159, osteoporosis 160 and bone tumors 161,162. Local minimally invasive drug injection for tumor treatment can maximise drug utilisation and cause less damage to other parts of the body. Based on this concept, Yan et al. 163 constructed mesoporous silica-coated gold nanorods (AuNR@SiO2) core-shell nanoparticles combined with GelMA and 5,6-dimethylxanthenone-4-acetic acid (DMXAA), in which the chemotherapeutic drug doxorubicin (DOX) and the osteosarcoma-targeting ligand alendronate were implanted. The nanocomposite system was found to significantly inhibit the growth of osteosarcoma, allowing for precise targeting of the treatment. Due to its good mechanical properties and biocompatibility, GelMA is often used in combination with a variety of chemotherapeutic agents to achieve sustained drug release for the treatment of osteosarcoma 164. The Sr2+ released from GelMA-based hybrid hydrogels can improve the proliferation and osteogenesis ability of stem cells and thus potentially treat bone tumor-related defects 165,166; photothermal therapy can promote the migration of metastatic bone tumors to cross-shaped microchannels embedded in GelMA hydrogel for therapeutic purposes 167; the photothermally controlled multifunctional implant (SP@MX/GelMA) can effectively kill osteosarcoma cells and bacteria while enhancing osteogenicity 162 (Figure 11). After bone tumor surgery, there are still multiple risks, including recurrence, infection and bone defects, so GelMA-based complexes with antibacterial and antitumor properties while filling bone defects are an important research direction. For instance, Yin, Jie et al. 162 developed a new multifunctional implant (SP@MX/GelMA) consisting of MXene nanosheets, GelMA hydrogel and bioinert sulphonated polyether ether ketone (SP), which can be used to promote tumor cell death, fight pathogenic bacteria and have osteogenic effects. In order to research the pathogenesis and treatment of tumors, 2D culture of tumor cells is now widely used, but it does not realistically mimic the environment. Based on this problem, He et al. 168 developed honeycomb GelMA hydrogel microspheres to construct a 3D model of osteosarcoma and found that this approach could better provide the physiological microenvironment of K7M2. It is evident that GelMA can be used not only for the treatment of bone tumors, but also as an effective strategy for the pathogenesis and drug screening.

## Molecular mechanism of GelMA-based materials for musculoskeletal diseases

**Figure 12:**
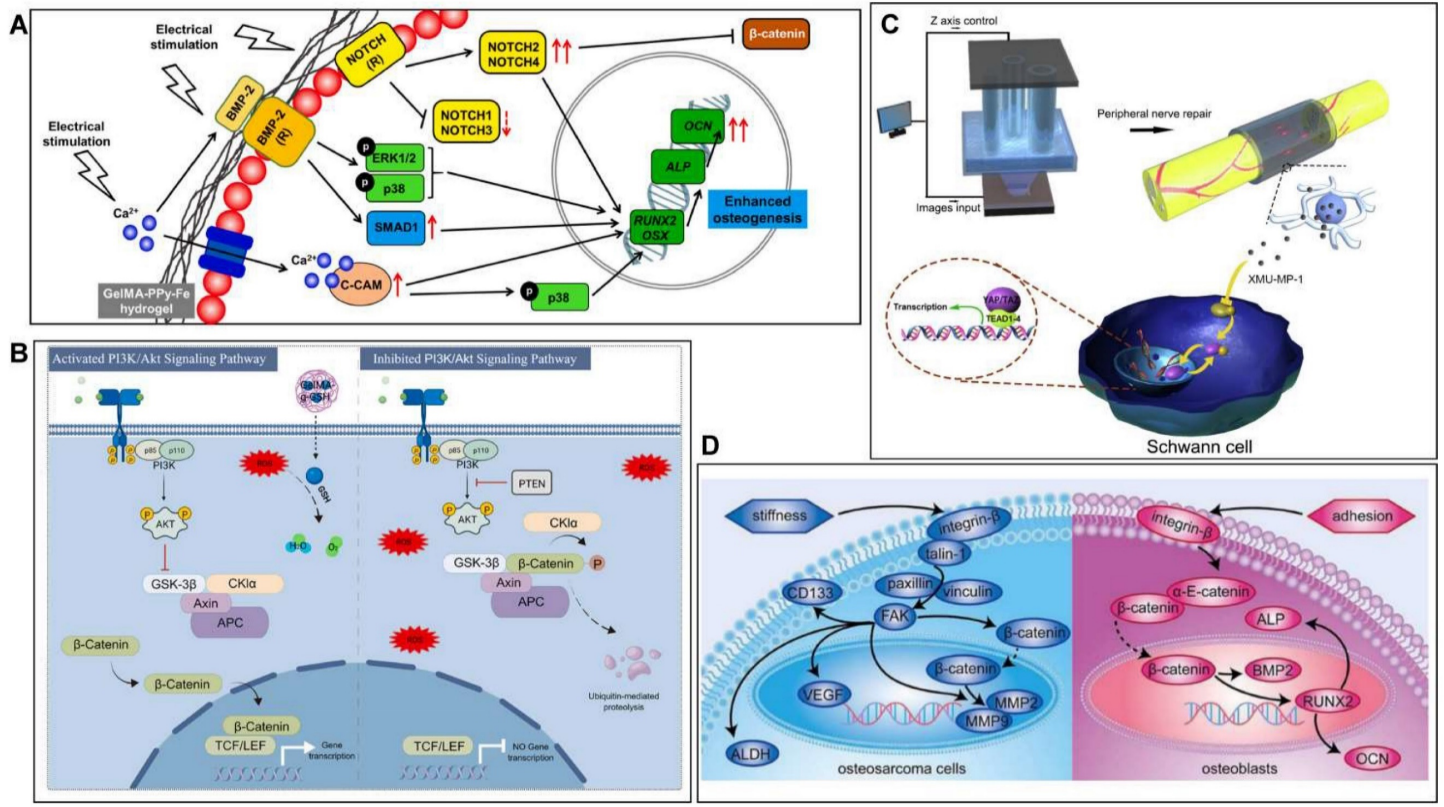
Molecular mechanism of GelMA-based materials for musculoskeletal diseases. **A**) GelMA-PPy-Fe promotes bone regeneration by regulating the NOTCH/MAPK/SMAD pathway and the Wnt/β-Catenin pathway. **B**) GelMA-g-GSH promotes bone regeneration through the PI3K/Akt signaling pathway. **C**) MPEG/PCL/GelMA mechanism for the treatment of PNI. **D**) Mechanism of PEGDA/GelMA in the treatment of bone tumors.

GelMA hydrogels have promising applications in the field of musculoskeletal repair because of their biodegradability, controlled mechanical properties and biocompatibility. Previous research on GelMA has focused on physical properties and chemical structure, but has neglected the molecular mechanisms that arise when it repairs musculoskeletal conditions. In recent years, with the rapid development of molecular biology, there has been an increase in research into the mechanisms by which bioengineered GelMA-based materials respond by interfering with multiple targets and pathways in the treatment of musculoskeletal disorders. For instance, Dutta et al. 169 constructed GelMA-PPy bioink by 3D bioprinting and demonstrated by transcriptome analysis that hBMSCs highly express NOTCH/MAPK/SMAD signalling while downregulating the Wnt/β-Catenin signalling pathway to promote osteogenesis (Figure 12A). Wang et al. 170 developed a reduced glutathione-grafted gelatin methacrylate (GelMA-g-GSH) antioxidant hydrogel by 3D printing technology to treat diabetic bone defects, which not only scavenges ROS but also activates the PI3K/AKT signalling pathway to promote osteogenesis and accelerate bone repair (Figure 12B). It was also found that the CPP-L/GelMA hydrogel constructed by Sun et al. 171 promoted bone regeneration through the Nrf2-BMAL1-autophagy pathway and was effective in scavenging ROS and regulating the bone microenvironment. Furthermore, the activation of signalling pathways such as YAP 172, HIF1-α 173 and BMP 174 is also an important molecular mechanism for GelMA hydrogels to promote bone regeneration. Cartilage defects are a common pathogenesis of OA, and the treatment of OA is mostly based on anti-inflammatory and promotion of cartilage repair. Yu et al. 175 showed that strontium ranelate (SrR) filled with silica nanospheres coupled with GelMA could promote cartilage differentiation and accelerate cartilage regeneration by inhibiting the Wnt/β-catenin signalling pathway. GelMA hydrogel-based materials can also significantly promote nerve regeneration when used in PNJ. As an example, Tao et al. 141 prepared GelMA/MPEG-PCL nerve conduits by 3D printing to restore nerve function and promote nerve regeneration through the release of Hippo pathway inhibitors. Bone tumors are also an important component of musculoskeletal disorders (Figure 12C). Jiang et al. 161 prepared PEGDA/GelMA hydrogels for the treatment of bone tumors and found that osteosarcoma cells modulate the FA pathway while osteoblasts modulate the AJ pathway when cultured in a 3D hydrogel matrix, which provides a basis for the study of anti-tumor approaches (Figure 12D). As GelMA hydrogels are progressively studied, it is crucial to understand their biological effects in musculoskeletal disorders (**Table 4**). Further exploration of their molecular mechanisms could provide a solid theoretical basis and scientific rationale for musculoskeletal regeneration.

## Clinical translations of GelMA hydrogels in musculoskeletal diseases

The numerous research examples mentioned above demonstrate the promising application of gelatin modified GelMA hydrogels in musculoskeletal diseases. Therefore, clinical translation is also one of the most urgent goals for GelMA hydrogels. Manferdini et al. 176 demonstrated through a systematic review the possibility of transferring cell-loaded hydrogels from research to clinical application, where they have shown a positive impact in the treatment of OA. In addition, they concluded that further research is needed on the properties of hydrogels, injection methods, adhesion strength, and chemotactic properties. In order to provide an injectable hydrogel with simple clinical application, Shi et al. 177 introduced nano-Silicate (SN) and stromal cell-derived factor-1α (SDF-1α) into GelMA hydrogel and the results demonstrated excellent injectability, superior osteogenic ability, promotion of stem cell homing and good controlled release, showing outstanding bone regenerative ability with easy clinical application.

For infected bone defects, clinical treatment often involves debridement and infection control followed by bone repair, but the results are not satisfactory. Therefore, Qian et al. 57 developed a new composite Ta/GelMA/PLGA/Van scaffold that has promising antimicrobial and bone regeneration capabilities and shows clear potential for clinical use. Recent studies have also found that engineered hydrogel microspheres for oxygen metabolic homeostasis (GM@CS-BP) can regulate ECM metabolic homeostasis in the myeloid nucleus and reduce the inflammatory response, providing a promising approach for the treatment of IVDD 99. In addition, a preclinical study has also demonstrated that GelMa hydrogels containing stem cells, manufactured using the ISC strategy, can improve muscle function and promote skeletal muscle regeneration for the treatment of Volumetric muscle loss (VML) 128.

Although there are many clinical applications and preclinical studies on hydrogels, hydroxyapatite and collagen are still widely used in bone, mainly due to the potential toxicity of cross-linking agents 178. Recently, Zhu et al. 179 showed that GelMA hydrogels combined with methacryloyl, and gelatin could achieve good batch-to-batch consistency in a highly controlled synthesis process and maintain good low cytotoxicity, which is crucial for clinical transformation. First of concern is biosafety of GelMA hydrogel and final degradation products, especially oligomeric methacrylates. An authoritative research shows that hydrogel obtained from the cross-linking of type-B (In the presence of a photoinitiator, after free radical generation, the methacrylamide and methacrylate pendant groups on the GelMA chain undergo radical addition polymerization to generate gelatin chains linked by short polymethacryloyl chains network) hydrogel shows good biosafety, while GelMA obtained from type-A hydrogel produces an obvious inflammatory reaction, which may be attributed to the endotoxin inherent in the latter material 180. The lack of pro-inflammatory activity leads to immunocompatibility of type-B GelMA hydrogels for the first time. Although the B-type GelMA hydrogel without endotoxin was used in this study, more studies are still carried out based on the type-A GelMA hydrogel, which has a higher level of endotoxin. These endotoxins may modulate favorable cellular behaviors (i.e., stimulate osteogenesis) or cause other adverse effects that obscure the observations 181. This aspect is often underestimated in the field.

Qualified standardization between batches of clinical translation and the possible immune response and disease transfer caused by materials are still huge challenges. Especially in groups with severe musculoskeletal diseases, people are more willing to try in exchange for possible health. Although GelMA has shown considerable promise in the treatment of musculoskeletal disorders, there are a number of hurdles to overcome before it can be used clinically. Firstly, the construction of the tissue material cannot be refined to meet the needs of the affected area, and the fabrication process for GelMA throws needs to be further improved. Second, the physicochemical properties of GelMA hydrogels are influenced by a variety of factors and the optimal environment for co-culture of multiple cells needs to be further explored. Thirdly, the immunological properties, mechanical properties, biocompatibility, bioconductivity, stability, degradation time, optimal time and dose of drug release, natural ECM environment and scaffold micropore size of GelMA are all directions that need further optimisation. Fourth, the way, method, dose and treatment time of hydrogel injection need further planning. Fifth, the current research is still stuck in cellular and animal experiments, animals are mainly carious animals, how to transition to primates, how to promote pre-clinical and clinical research safely and effectively, how to develop uniform standards for clinical application, and how to carry out good clinical translation are all issues that need to be explored. It is clear that the clinical use of GelMA in musculoskeletal disorders still needs a long road to validation, and further optimisation of its biological characteristics and reasonable and safe operational procedures is urgent.

## Conclusion and outlook

We summarize applications of GelMA-based hydrogel systems in the field of musculoskeletal tissue engineering according to the following characteristics. (1) ECM-mimic structure; (2) RGD peptide sequence improve cell adhesion; (3) MMP degradable motifs and excellent biocompatibility.

GelMA-based hydrogels have emerged as an exciting potential method for musculoskeletal tissue engineering and have shown considerable promise. Strategies using GelMA-based hydrogels retain biocompatibility and bioactivity of gelatin and render them potentially ideal repair methods.

Compared to unfunctionalized platforms, certain nanocomposite hydrogels could be applicable only in culturing specific cell types, due to the intrinsic material characteristics. The incorporation of synthetic and natural substrates modifies the mechanical properties of GelMA. Hence, developed methods offer opportunities to adopt GelMA-based hydrogels to control sustained release systems in various biological fields, including repair or regrowth of specialized connective tissues (cartilage, bone, skin, tendon, muscle, spinal cord, and nerve tissues) as well as capability in inducing cell recruitment, differentiation, angiogenesis, and anti-apoptosis.

GelMA hydrogels are much like ECM, which is the basis feature of future applications. For instance, the RGD-containing motifs facilitate cell attachment and proliferation, and the MMP-containing motifs participate in cytoskeletal remodeling. The containing motifs offer tissue-mimicking 3D microenvironment to secure survival and promote adaptations to target tissue. Their potential therapeutic usage was confined to constructing specific tissue analogs due to weak mechanical properties. The properties of GelMA hydrogels have been modified by many technologies to achieve excellent performance in various tissue engineering applications. Notably, hydrogels loaded with natural or synthetic agents, such as incorporating bioactive agents (nanoparticles, growth factors, extracts, antibiotics, etc.) into the local lesions.

Considerable progress in GelMA has been realized, there are still several aspects to be noted when moving towards clinical translation. Future clinical applications are now focusing on the combining of various cell-types and different microencapsulation techniques to work in a complementary mode. In addition, chemotherapy, gene, and photothermal therapy treatment maximize the therapeutic results in combination with GelMA implantation. To conclude, GelMA-based biomimetic scaffolds will act as pioneers in biomedical applications which remain to be explored.

## Figures and Tables

**Table 1:** Representative application of GelMA in bone regeneration.

Materials/Hydrogels	Fabrication methods	Cell type	Animal species	Research stages	Outcomes	References
GelMA/ Mg-IHC	Physical crosslinking/atomization	MC3T3-E1	Mouse	*In vitro*/Animal	Promote bone regeneration	38
GelMA/ nHAp	Chemically crosslinking using EDC/NHS	MC3T3 E1	-	*In vitro*	Minimally invasive therapy for bone regeneration	39
Gel-GNP	Chemical crosslinking/ photocrosslinking	MC3T3-E1/ADSCs	Rabbit	*In vitro*/Animal	Promote proliferation, viability, and osteogenic differentiation of ADSCs	40
GelMA/ Nanosilicate	Photopolymerization	MC3T3	-	*In vitro*	Favorable mechanical properties, osteogenic capability in absence of inducer	41
GelMA/2D-nSi	Physical crosslinking/photocrosslinked	hMSCs	Rat	*In vitro*/Animal	ECM mimicking scaffolds promote osteogenic differentiation in absence of growth factor	42
GelMA/nHA/PLLA	Photocrosslinked/ electrospinning	hBMSC	Rat	*In vitro*/Animal	Promote hBMSC proliferation, adhesion, and osteogenic differentiation	43
GelMA/ Ti-6Al-4V	Physical crosslinking	3T3 fibroblast cells	-	*In vitro*	Increase the specific surface area and enhance the implant surface coverage	44
GelMA/ PCL	Physical crosslinking /photocrosslinked/3D Shape	hNSCs	-	*In vitro*	Exhibit ordered structures and repair the damaged tissues	45
GelMA/ GA	Physical crosslinking/photocrosslinked	hBMSCs	Mouse	*In vitro*/Animal	Promote osteogenic differentiation and enhance calcium deposition, promote bone fracture healing	46
GelMA/ tofu	Physical crosslinking/ photopolymerization	hDPSCs/macrophage	-	*In vitro*	Increase the secretion of osteogenesis and immune-related cytokines and hold enormous therapeutic potential for bone regeneration	47
GelMA/MA-CS-GMA	3D Printing	DPSCs	-	*In vitro*	Exhibis excellent biocompatibility, increase proliferation of stem cells, promote the matrix mineralization	48

**Table 2:** Advances of GelMA in skeletal muscle engineering.

Polymer scaffold	Fabrication methods	Cell	Research stages	Outcomes	Reference
GelMA/CMCMA GelMA/AlgMA	3D bioprinting	C2C12	*In vitro*	Show high cell viability, proliferation, myogenesis, and structural stability	117
GelMA	microfluidic device	C2C12	*In vitro*	Show excellent cell supportive properties to induce myoblast alignment and enhance myotube formation and maturation	118
GelMA	geometrical confinement	C2C12	*In vitro*	promote myogenic precursors differentiation	119
GelMA	the pillar well-array based stretching device	C2C12	*In vitro*	promote the myoblasts differentiation and contractility of myofibers	120
GelMA/Laponite nanoclay	Physical crosslinking	-	Animal study	promote muscle recovery, inhibit fibrosis, and increase anabolic response	121
GelMA/NaA	microfluidic device	C2C12	*In vitro*	Exhibit excellent orientational function and biocompatibility	122
GelMA-AuNPs GelMA-MXene	Bioprinting	C2C12	*In vitro*	enhanced differentiation of encapsulated myoblasts	123
GelMA-GO	3D bioprinting	PC-12	*In vitro*	Excellent conductive properties	124

**Table 3:** The recent application of GelMA in nerve regeneration engineering.

Polymer scaffold	Fabrication methods	Cells	Research stages	Outcomes	References
GelMA/PGFs	a melt quenching and fiber drawing method	C6	*In vitro*	promote directional growth of glial cells	133
GelMA/MeHA	Physical crosslinking /photocrosslinked/3D printing	Schwann cells/PC-12	*In vitro*	conducive for neural cell infiltration, cell adhesion, and growth factor sequestration	134
GelMA	Physical crosslinking /photocrosslinked	iNSCs	*In vitro*/Animal	inhibit GFAP-positive cells and glial scar formation while promoting neurite regrowth	135
GelMA/EHS	3D printing	Neuron	Animal	bridg nerve defects and promote the repair of peripheral nerve	136
GelMA/PCL/rGO	Physical crosslinking/electrospinning	RSC96	*In vitro*/Animal	promote motor/sensory nerve regeneration and functional recovery	137
r(GO/GelMA)	Chemically crosslinking using EDC/NHS	PC12	*In vitro*/Animal	promote neural regrowth, synaptic regeneration, myelin formation, and dendritic branch formation	138
GelMA	Photopolymerization (315-400 nm)	Neuron	Animal	inhibite scar formation and cure syringomyelia	139
GelMA/Ni/GMT Ca2+-Alginate	Physical crosslinking /photocrosslinked/ chemical vapor deposition	RSC96	*In vitro*/Animal	facilitate peripheral nerves restoration and denervated muscle regeneration	140
GelMA/MPEG-PCL	Physical crosslinking /photocrosslinked	Schwann cells	*In vitro*/Animal	facilitate restoration of nerve injuries in morphology and functions	141
GelMA/bFGF	Physical crosslinking /photocrosslinked	DPSCs	*In vitro*/Animal	treat the peripheral nerve injuries with large nerve deficits	142
GelMA-DA	Physical crosslinking /photocrosslinked/3D printing	NSCs	*In vitro*	promote neural differentiation	9

**Table 4:** The recent molecular mechanisms of GelMA-based materials for musculoskeletal repair.

Polymer scaffold	Disease	Cells	Animals	Research stages	Mechanism	References
GelMA-PPy-Fe	Bone defect	hBMSCs	-	*In vitro*	NOTCH/MAPK/SMAD pathway; Wnt/β-Catenin pathway	169
GelMA-g-GSH	Bone defect	MC3T3-E1	Mouse	*In vitro/In vivo*	PI3K/Akt pathway	170
CPP-L/GelMA	Bone defect	MC3T3-E1, RAW264.7, HUVEC	Mouse	*In vitro/In vivo*	Nrf2-BMAL1-autophagy pathway	171
GelMA/dextran emulsion	Bone defect	BMSCs	Rat	*In vitro/In vivo*	YAP pathway	172
GelMA/GGMA	Bone defect	HUVECs, BMSCs	Rat	*In vitro/In vivo*	HIF1-α pathway	173
SiGO/GelMA	Bone defect	hMSCs	Mouse	*In vitro/In vivo*	BMP-SMAD1/5 pathway	174
SrR/GelMA	OA	BMSCs	Rat	*In vitro/In vivo*	Wnt/-catenin pathway	175
MPEG/PCL/GelMA	PNI	S16, HUVECs	Rat	*In vitro/In vivo*	Hippo pathway	141
PEGDA/GelMA	Bone tumor	MG63, hFOB1.19	Mouse	*In vitro/In vivo*	FA and AJ pathway	161
